# A New Paradigm for Known Metabolite Identification in Metabonomics/Metabolomics: Metabolite Identification Efficiency

**DOI:** 10.1016/j.csbj.2015.01.002

**Published:** 2015-01-27

**Authors:** Jeremy R. Everett

**Affiliations:** Medway Metabonomics Research Group, University of Greenwich, Chatham Maritime, Kent ME4 4TB, United Kingdom

**Keywords:** Metabolite identification efficiency (MIE), Metabolite identification carbon efficiency (MICE), Metabonomics, Metabolomics, NMR spectroscopy, Molecular spectroscopic information

## Abstract

A new paradigm is proposed for assessing confidence in the identification of known metabolites in metabonomics studies using NMR spectroscopy approaches. This new paradigm is based upon the analysis of the amount of metabolite identification information retrieved from NMR spectra relative to the molecular size of the metabolite. Several new indices are proposed including: metabolite identification efficiency (MIE) and metabolite identification carbon efficiency (MICE), both of which can be easily calculated. These indices, together with some guidelines, can be used to provide a better indication of known metabolite identification confidence in metabonomics studies than existing methods. Since known metabolite identification in untargeted metabonomics studies is one of the key bottlenecks facing the science currently, it is hoped that these concepts based on molecular spectroscopic informatics, will find utility in the field.

## Graphical abstract

**Figure:**
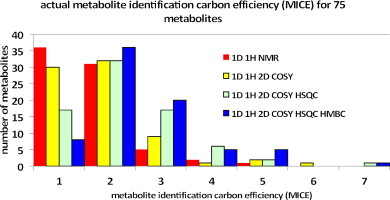

## 1 Introduction

Metabonomics is defined as ‘The study of the metabolic response of organisms to disease, environmental change or genetic modification’ [1] and has emerged as a leading technology in a number of fields, including biology and medicine [2], with new areas emerging recently, such as pharmacometabonomics for personalised medicine [3–5]. The alternative term metabolomics [6] was defined a little later as a ‘comprehensive analysis in which all the metabolites of a biological system are identified and quantified’. The two terms are now used interchangeably but in this article we will refer to the original term throughout.

Metabonomics studies are typically conducted with either nuclear magnetic resonance (NMR) spectroscopy or a hyphenated mass spectrometry (MS) technology, such as liquid chromatography–MS (LC–MS) [7], to acquire information on the identities and quantities of metabolites in the particular samples of interest. The studies are conducted either in a targeted fashion, where a pre-defined set of metabolites are measured, or in an untargeted fashion, where no preconceptions of the metabolites of importance are imposed. The choice of analytical technology used often depends upon the particular study requirements.

In this article, the focus will be on the use of NMR spectroscopy rather than MS, although the two technologies are quite complementary and it is often advantageous to use them together in concert.

Metabonomics/metabolomics studies have a number of important elements including:

1. definition of study aims e.g., understanding the metabolic consequences of disease progression in a particular group of patients
2. ethical approval
3. sample collection and storage
4. sample preparation
5. NMR data acquisition
6. quality control of the acquired data to ensure adequate signal-to-noise, lineshape and resolution
7. spectroscopic data pre-processing steps such as zero-filling, apodisation, Fourier transform, phasing and baseline correction
8. statistical data pre-processing steps such as peak alignment, scaling and normalisation of the data
9. statistical analysis of the data to interrogate differences between groups of subjects e.g., healthy volunteers vs patients with disease
10. identification of metabolites responsible for any inter-group differences discovered in the study
11. rationalisation of the role of the discriminating metabolites in terms of physiological and biochemical changes in the subject.

Many of the 11 steps above have been subject to rigorous study and guidelines have emerged for several areas including NMR-based sample preparation, data acquisition, data pre-processing and statistical analysis of the data, especially by multivariate methods [8–15]. However, the critical step for many untargeted metabonomics studies is the identification of the metabolites that are responsible for discriminating between different groups of subjects in the study: step 10. This remains problematical for both MS [16,17] and NMR spectroscopy [14,18–24] and is a significant bottleneck for the development of the science.

The issue with metabolite identification was nicely illustrated by Wishart who contrasted the 4 different bases in the human genome, and the 20 natural amino acids in the human proteome, with the thousands of different metabolites in the human metabolome: this is the cause of the issue [25].

For studies where many samples are available, statistical methods of metabolite identification, such as STOCSY and variants thereof, are powerful tools that can be used for metabolite and biomarker identification [26,27]. In genome wide association studies on metabonomics data, the pathway information that can be gleaned can also be used to help identify key metabolites, including by metabomatching [28,29].

Metabolite identification by NMR spectroscopy has recently been significantly facilitated by the development of spectral databases of metabolites [30], such as the Human Metabolome Database (HMDB) [31], the BioMagResBank (BMRB) [32] and the Birmingham Metabolite Library (BML) [33]. These libraries not only store information on the NMR spectra of a vast array of metabolites, which helps metabolite identification, but more powerfully, some also allow downloading of the original NMR free induction decay data from the databases, to facilitate comparison of the spectral features of authentic metabolites with those of unidentified metabolites in users' biological samples.

Some progress has been made towards the automated identification of metabolites but these methods are not yet at the stage that they can be routinely used to identify more than a fraction of the metabolites in complex biofluids such as urine. The Birmingham Metabolite Library (BML) provides a facility for the matching of experimental 2D ^1^H J-resolved spectra with those of reference metabolites stored in a database, which is a good approach, but is limited by the low number of metabolites in that database [33]. Approaches such as MetaboHunter have been applied to the identification of mixtures of standard compounds but not to a biofluid [21]. An approach based on 1D ^1^H NMR profiles, has had success in identifying metabolites in human serum and cerebrospinal fluid, but was less successful in identifying metabolites in urine due to spectral complexity and the lack of a complete reference set [34]. A different 1D ^1^H NMR approach based on extraction of relevant variables for analysis (ERVA) has been applied to simulated mixtures and to the analysis of tomato extracts, but again relies on the availability of authentic spectra of the metabolites and fails for compounds that have only a single peak in their 1D ^1^H NMR spectrum [24]. Thus, at the present time, the only robust way to identify known metabolites in the 1D ^1^H NMR spectra of complex biofluids such as urine is by manual analysis by an expert NMR spectroscopist.

The metabolite identification issue is in two distinct categories: first the *structure elucidation* of truly novel metabolites, not previously reported, and secondly, the *structure confirmation* or *structure identification* of previously reported or known metabolites. This simple language and description is consistent with decades of molecular structure elucidation literature, and is preferable to the more complex and confusing labelling of metabolites as ‘unknown unknowns’ or ‘known unknowns’ that has emerged more recently [25,35]. For the structure elucidation of truly novel metabolites, there is a consensus that the same rigorous processes used in the natural product field should be adopted in metabolite identification. This would usually involve extraction and purification of the novel metabolite, followed by full structure elucidation by ultraviolet, infrared and NMR spectroscopies in concert with MS [36,37].

However, the process of structure confirmation of known metabolites remains an issue, due to differences in approaches across the metabonomics/metabolomics community. In order to address the metabolite identification issue, the Metabolomics Standards Initiative (MSI) [38] set up a Chemical Analysis Working Group (CAWG) which proposed a 4-level classification system (Table 1) for the structure confirmation of known metabolites in 2007 [36].

In the seven years since these proposals were made, they have not been widely adopted by the community [39]. There are two basic problems with the original proposals: firstly, the requirement of comparison of experimental data for known metabolites to an authentic reference standard in the lab, is often too strict and not always appropriate for an NMR-based study, and secondly, the system is too coarse and does not define closely enough the confidence achieved in the metabolite identification. Recently, new proposals emerged to update the 4-level system with either: (i) addition of sub-levels to grade confidence better, (ii) an alternative quantitative identification points scoring system or (iii) quantitative enhancement of the current 4-level system to indicate confidence [40]. An overlapping subset of the same authors also proposed quantitative and alphanumeric metabolite identification metrics [41]. The quantitative scoring proposal in the latter publication contains a mixture of excellent metabolite identification criteria with precision e.g., accurate mass of parent ion (< 5 ppm) and processes such as having a COSY NMR, with no precision or scoring for matching. It was commented that it is difficult to see how scoring for matching of metabolite data to standards could be achieved [41]. A call to the community was made for engagement with this important problem [40].

This paper responds to those calls. A new approach to the understanding of the NMR spectroscopic information theoretically embedded in metabolites is put forward, and compared with the data that is actually obtained in the course of metabonomics experiments. Conclusions and proposals are arrived at in terms of a different approach to metabolite identification confidence, which should be applicable in spirit to any other analytical technology, in addition to NMR spectroscopy.

## 2 Material and methods

### 2.1 Subjects, sample preparation and NMR spectroscopy

The 75 metabolites included in this study were identified manually from the proton NMR spectra of the urine from a C57BL/6 mouse at 30 weeks of age, and the urine of a diabetic patient on an exercise study at La Sapienza University, Rome. Both studies were ethically approved [42].

The diabetic urine sample was prepared by mixing urine (630 μl) with phosphate buffer (70 μl of an 81:19 (v/v) mixture of 1.0 M K_2_HPO4 and 1.0 M NaH_2_PO4 pH 7.4). After standing at room temperature for 10 min, the sample was centrifuged at 13,000 *g* for five minutes at 4 °C to enable separation of clear supernatant (600 μl) from any particulate matter. The supernatant was mixed with a solution of the chemical shift reference material, sodium 3-(trimethylsilyl) propionate-2, 2, 3, 3-d4 (TSP) in D_2_O (60 μl), to give a final TSP concentration of 0.18 mM.

The mouse urine sample was prepared by mixing pooled urine (500 μl) from a single C57BL/6 mouse with phosphate buffer (150 μl of a 81:19 (v/v) mixture of 0.6 M K_2_HPO4 and NaH_2_PO4 in 100% ^2^H_2_O, pH 7.4, containing 0.5 mM TSP as a reference and 9 mM sodium azide). The sodium azide was added to prevent bacterial growth in the urine sample.

All NMR experiments were conducted on a Bruker Avance spectrometer operating at 600.44 MHz for ^1^H NMR, at ambient temperature, in 5 mm NMR tubes (508-UP-7). All chemical shifts are on the δ_H_ or δ_C_ scales relative to TSP at 0.

The identification of the metabolites used a combination of standard 1D and 2D NMR methods, including J-resolved (JRES), COSY, TOCSY, HSQC and HMBC experiments. The 1D ^1^H NMR experiments used the 1D NOESY presaturation pulse sequence, noesypr1d. Free induction decays were collected into 65,536 data points with 256 scans and 4 dummy scans and a spectral width of 12,019.2 Hz. The resulting spectra were zero-filled to 131,072 or 262,144 points, baseline corrected automatically, phase corrected automatically (with manual override, as required) and apodised for resolution enhancement using Gaussian multiplication. The detailed parameters for the acquisition of the 2D NMR spectra are given in Supplementary Table 1.

### 2.2 Theoretical analysis of the NMR spectroscopic information content of the 75 metabolites

All 75 metabolites were characterised by their common names, IUPAC name, HMDB code, SMILES string, InChi code and InChi key (see Table 4 and Supplementary Data). 14 Molecular features were analysed manually for each of the 75 metabolites represented in this study (Table 2):

The following rules were applied to this feature analysis:

1. Only non-exchanging protons were included in the analysis of the number of proton chemical shifts present in the metabolites, and this included non-exchanging (on the NMR timescale) amides but not hydroxyl, amine or acid protons
2. The number of multiplicities is simply the sum total of the number of singlets, doublets, doublet of doublets etc. contained in a metabolite: for example, if a metabolite has one singlet and two doublet proton signals, the multiplicity count for that metabolite is three
3. The total number of coupling constants was calculated for all possible 2- and 3-bond proton-to-proton couplings involving non-exchanging protons
4. COSY cross-peaks between two protons were only counted once: therefore the number of COSY peaks must equal the number of coupling constants: long-range COSY connectivities were not counted
5. All HSQC cross-peaks including those from non-equivalent methylene protons on the same carbon were counted. However, symmetrically-equivalent HSQC or HMBC cross-peaks, such as those that occur in succinic acid for example, were counted only once: the analysis reflects the number of peaks that can be seen in the spectra.
6. The count of theoretical ^1^H, ^13^C HMBC NMR cross-peaks includes all possible 2- and 3-bond carbon-to-proton couplings, including those between pseudo-equivalent groups e.g., the methyl groups in trimethylamine, as these are real and provide useful information for the identification of small metabolites
7. The second-order flag was only set in cases where the presence of magnetically non-equivalent but chemically equivalent protons would give rise to additional transitions in the spectra, not merely for cases where the signals have intensity distortions. The flag is set to 1 if there are ≥ 1 of these second order features and 0 otherwise.
8. For sugars, the count of features is applied to *both* anomers.

Thirteen parameters, A to M, were then calculated from the 14 features (Table 3): see also the Supplementary Data.

### 2.3 Analysis of the NMR spectroscopic data and metabolite identification

All spectral processing was conducted in MNova version 9.0.0-12821 (Mestrelab Research S.L.).

Analysis of the spectroscopic information content of the urinary metabolites was conducted manually and captured and further analysed in Excel for Mac 2011 version 14.4.6 (Microsoft Corporation). Student t-tests were run in Excel using 2-tailed, unpaired calculations to determine the statistical significance of differences in values between groups of data. A p value of < 0.05 was used as the cut-off for statistical significance [43]. IUPAC names, SMILES strings and InChi codes for the metabolites were downloaded from either the Human Metabolome Database [31] or from ChemSpider (Royal Society of Chemistry). Although 2D ^1^H JRES NMR gives no new information (except for 2nd order systems and the discrimination of homonuclear and heteronuclear coupling), it was used to assist with the analysis of the 1D ^1^H NMR spectra, and some coupling and multiplet information was abstracted from 2D ^1^H JRES NMR rather than the 1D ^1^H NMR spectra if appropriate. Similarly, TOCSY data was occasionally used to assist spectral analysis in crowded regions, although it theoretically provides no new information over COSY in the absence of spectral crowding.

Features numbered 8 to 14 in Section 2.2 above were then analysed in the *actual* 1D and 2D NMR spectra of the urines of the mouse and diabetic patient, as features 8′ to 14′ respectively. For example, the total number of ^1^H NMR chemical shifts (8′) actually observed for each metabolite (as opposed to the theoretical number calculated in Section 2.2 above) was measured. Parameters equivalent to B to M in Section 2.2 above were then calculated from features 8′ to 14′ to give the values for B′ to M′ respectively, for comparison with the theoretical values. For instance, the *actual* metabolite identification carbon efficiency (MICE) for 1D ^1^H NMR level data is parameter F′. The full spreadsheet containing all these data is available as Supplementary Data.

## 3 Results and discussion

### 3.1 The identification of 75 human and mouse urinary metabolites

The manual analysis of a range of 1D and 2D NMR spectra of mouse and human urines from two recent studies [42] had resulted in the identification of a total of 75 metabolites. These metabolites were identified on the basis of NMR spectral analysis and comparison of the spectral data of the metabolites with that available for standard reference metabolites in the Human Metabolome Database [31], the BioMagResBank (BMRB) [32] and the Birmingham Metabolite Library (BML) [33]. The exact methodology for the analysis will not be detailed here but typically involved: (i) comprehensive comparison of the 2D ^1^H, ^13^C HSQC data with reference data in the HMDB and (ii) further interrogation of the data using all available resolution-enhanced 1D ^1^H and 2D ^1^H JRES, COSY, TOCSY and HMBC data.

**Fig. 1:**
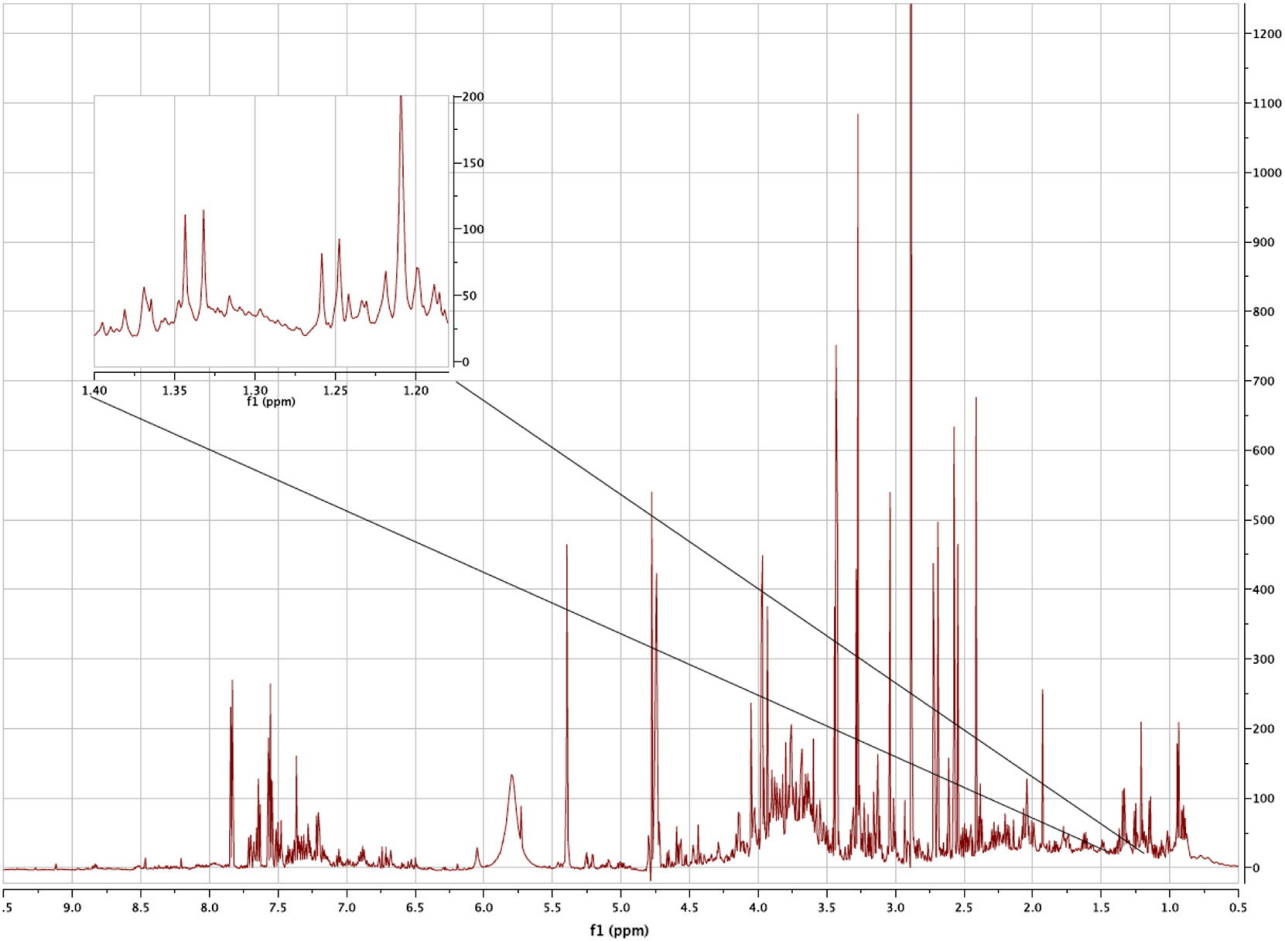
The 600 MHz ^1^H NMR spectrum of the urine from a C57BL/6 mouse and an expansion in the region of the methyl signals from lactic acid and the two anomers of L-fucose. The spectrum is moderately resolution-enhanced by Gaussian multiplication.

As an example, the alpha and beta anomers of L-fucose (6-deoxy-l-galactose), a methyl sugar, were identified in the mouse urine. The process of this identification is described here. The 600 MHz 1D ^1^H NMR spectrum of the mouse urine is shown in Fig. 1. Hundreds of signals are seen for dozens of metabolites. The identification of the known metabolite L-fucose commenced by matching the cross-peak at 1.25, 18.47 in the HSQC spectrum (Supplementary Fig. 1) to the methyl group of the beta anomer of L-fucose by an HMDB 2D HSQC search. The database gives figures of 1.26, 18.3 for the beta anomer of L-fucose. In confirmation, the ^3^J_H,H_ coupling constant of the doublet at ca 1.25 in the mouse urine was measured as 6.5 Hz, in accordance with the HMDB figure. Naturally, if the beta anomer of L-fucose is present, then the alpha anomer must also be detected, as they are in dynamic equilibrium, although it is expected to be present at lower levels.

**Fig. 2:**
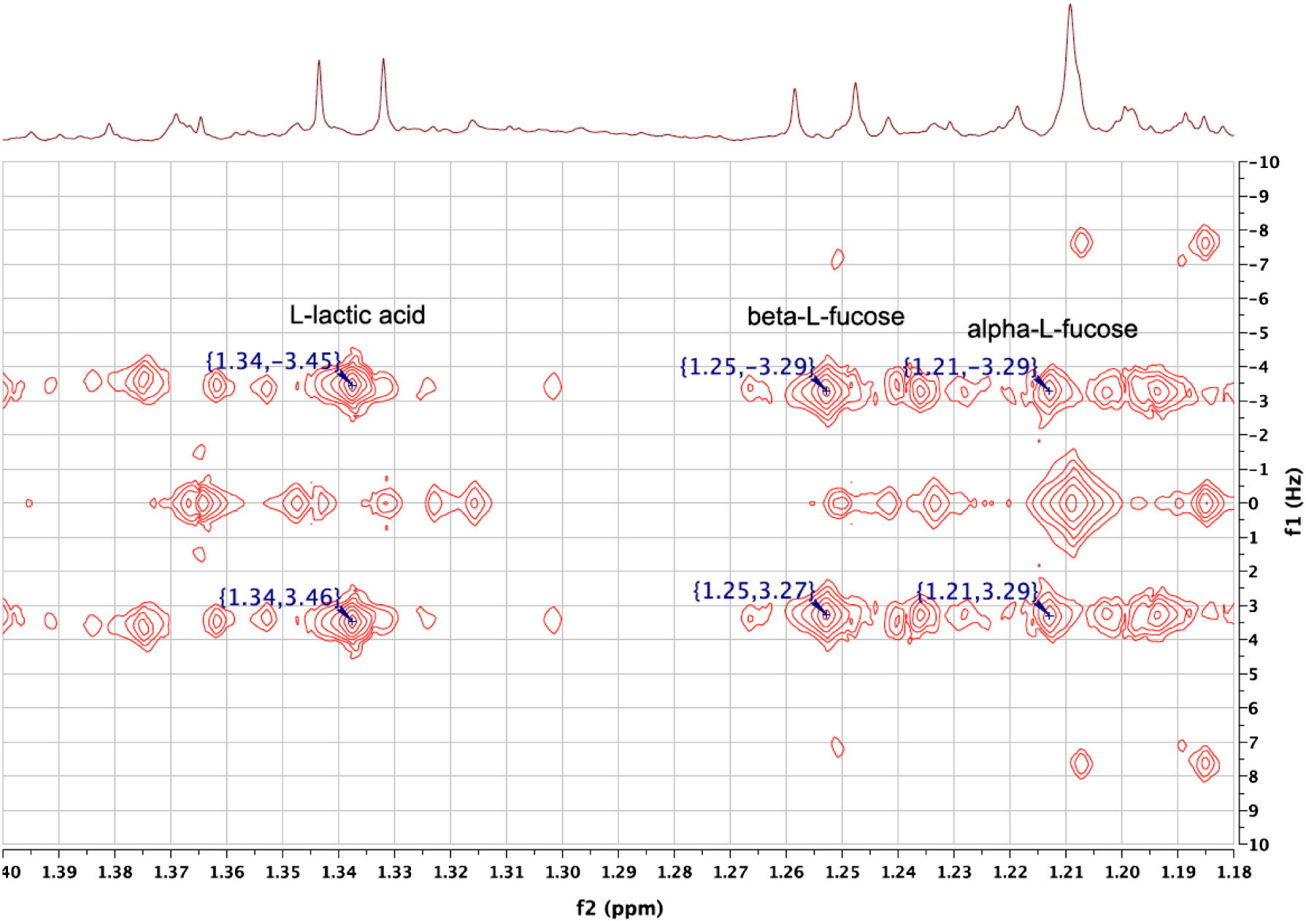
An expansion of the 600 MHz 2D ^1^H J-resolved NMR spectrum of the urine from a C57BL/6 mouse in the region of the methyl signals from lactic acid and the two anomers of L-fucose, underneath the corresponding region of the 1D ^1^H NMR spectrum.

No signals for the alpha anomer were clearly visible in the 1D ^1^H NMR spectrum, but the HSQC spectrum displayed a cross-peak at 1.22, 18.4, which corresponded well with the HMDB data for the authentic reference material (1.20, 18.3). The ^3^J_H,H_ coupling constant between the alpha methyl and H5 was 6.6 Hz, in good agreement with HMDB (6.7 Hz). This was measured in the 2D J-resolved ^1^H NMR spectrum (Fig. 2), where the hidden alpha anomer signal is revealed by the spreading-out of the overlapped metabolite signals across a second dimension.

Further confirmation that these signals belonged to L-fucose came from a 2D ^1^H COSY NMR spectrum (Supplementary Fig. 2), which showed that the methyl doublets resonating at ca 1.25 and at ca 1.21 in the mouse urine spectrum were connected to protons resonating at 3.80 and 4.20 respectively, exactly as expected for the beta and alpha anomers of l-fucose according to HMDB00174, which gives 3.80 and 4.18 respectively. A 2D ^1^H, ^13^C HMBC NMR spectrum also showed that the methyl protons at ca 1.25 connected to a carbon resonating at 73.7, which is a good match for C5 in beta-l-fucose (HMDB00174 gives 73.5).

In addition to these data, signals for the alpha and beta anomeric protons of l-fucose were detected at 5.22 (doublet (d), ca 4.0 Hz, COSY to H-2 at ca 3.78, HSQC to 95.5) and 4.57 (d, ca 7.8 Hz, COSY to 3.46, HSQC to 99.5) respectively (data not shown). Interestingly, the latter COSY revealed that there had been a data misinterpretation in HMDB (HMDB00174, accessed 29th November 2014) as the resonance for H-2 beta is given as 3.64 instead of 3.46, even though the coupling data matches the signal reported in HMDB at 3.46 and not at 3.64. HMDB00174 gives 5.19 (d, 3.9 Hz), 95.1 and 4.54 (d, 7.9 Hz), 99.0 for the anomeric protons and carbons of the alpha and beta anomers respectively.

It seemed that the metabolite whose signals were observed in the mouse urine was definitely l-fucose. However, according to the MSI guidelines, the identification could only be classified as a putative annotation, as the comparisons were made relative to the data in the HMDB, rather than to an authentic reference standard (Table 1). Indeed, all 75 metabolites identified in the studies of the mouse and human urine (Table 4) could only be described as putatively annotated by these rules. This seemed inappropriate and unsatisfactory, as the confidence in the identification of the vast majority of these metabolites was very high. It seems that the MSI 4-Level system is too conservative for metabolite identification based on NMR spectroscopic data, which in comparison to chromatographic retention time data, or electrospray MS signal intensity data, for example, is more predictable and precise.

In order to explore what information had been acquired and how it compared with what was theoretically available, an analysis of the spectroscopic information present in the 75 metabolites identified in the two metabonomics studies was undertaken.

The 75 metabolites from the two studies were combined to provide a realistic representation of the range of metabolites that a typical metabonomics study by high field NMR might identify. Analysis of these molecules showed that they had a molecular weight range of 31 to 284 Da (nominal mass) with an average of 126.7 ± 46.6 Da. The number of carbon atoms ranged from 1 to 13 with an average of 4.9 ± 2.2 (standard deviations). See the Supplementary Data for more information.

The subsequent analysis was completed in three parts: (i) an analysis of the information content of 1D and 2D ^1^H NMR spectra; (ii) an analysis of the NMR spectroscopic features theoretically present in the 75 metabolites and (iii) a comparison of the features theoretically present in the metabolites with those actually found in the course of the metabonomics studies. The aim of these analyses was to determine how much structural information was present in the metabonomics data and therefore how much confidence could be ascribed to metabolite identification. This analysis proved to be both informative and thought provoking.

### 3.2 The Information content of NMR spectra in the context of metabonomics experiments

NMR spectroscopy provides a surprisingly rich quantity of information on the molecules under study. The following list of 11 NMR spectral features is not exhaustive but includes those that are useful for the purposes of metabolic profiling, and is focused on ^1^H NMR-detected experiments: (1) chemical shifts, (2) signal multiplicities, (3) coupling constants, (4) 1st or 2nd order signal nature, (5) signal half-bandwidth, (6) signal integral, (7) COSY cross peaks, (8) HSQC cross-peaks, (9) HMBC cross-peaks, (10) TOCSY cross-peaks and (11) signal rate of change [44]. A detailed analysis of these features is provided in Supplementary Table 2.

### 3.3 Analysis of the theoretical 1D and 2D ^1^H NMR spectroscopic information content of metabolites

Of the 11 features of 1D or 2D ^1^H NMR spectroscopy outlined in Section 3.2 above, the analysis here focused on just 7: chemical shifts, multiplicities, coupling constants, 2nd order nature and COSY, HSQC and HMBC cross-peaks, for further study, as these are of most importance for metabolite identification by NMR spectroscopy. A manual analysis of the *number* of *each* of these 7 features expected to occur in *each* of the 75 metabolites was conducted (see Supplementary Data).

Table 5 shows the number of bits of spectroscopic information theoretically present in the 1D or 2D ^1^H NMR spectra of the 75 metabolites, for a range of different approaches to metabolite identification. The first would involve just the use of 1D ^1^H NMR; the second, the additional use of COSY, the third the additional use of HSQC and finally, the additional use of HMBC information. For example, the number of bits of spectroscopic information in a metabolite for an approach based on just 1D ^1^H NMR would include the total number of ^1^H NMR chemical shifts, multiplicities and coupling constants in a metabolite, plus a flag for a second order spin system, if present.

**Fig. 3:**
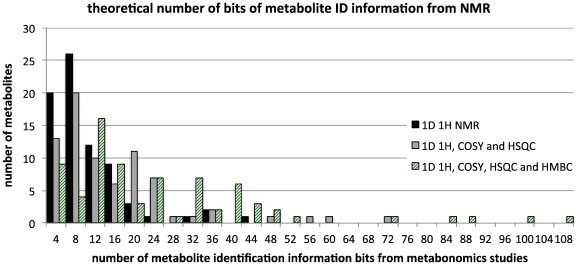
The distribution of the theoretical number of bits of metabolite identification (ID) information available from three different NMR approaches across the 75 metabolites. The number of bits is calculated in bins ranging from 0 to 4, 5 to 8 etc. up to 105 to 108 bits. Each bit represents a ^1^H NMR chemical shift, multiplicity, coupling constant, 2nd order flag, COSY cross-peak, HSQC cross peak or HMBC cross peak, that theoretically should be observed for the metabolite in question. Data for approaches using ^1^H plus COSY data not shown for clarity of presentation.

Thus, as expected, the amount of spectroscopic information available to assist with metabolite identification increases in going from approaches based solely on 1D ^1^H NMR, to those involving significant utilisation of 2D NMR methods. The distribution of the data across the 75 metabolites is informative (Fig. 3). *The bits of spectroscopic information can be considered to be bits of metabolite identification information*, *each of importance to the valid identification of metabolites*. What is immediately apparent is that even with a simple 1D ^1^H NMR approach, some metabolites contain a surprisingly large number of bits of information that can be used to identify them: up to 42 bits in one metabolite in this set.

**Fig. 4:**
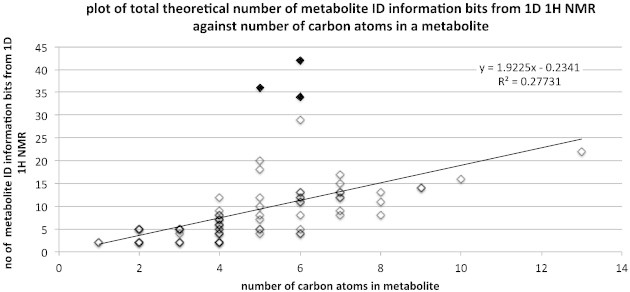
The number of metabolite identification (ID) information bits theoretically available from 1D ^1^H NMR plotted against the number of carbon atoms for all 75 metabolites. Three outliers due to xylose, fucose and glucose are highlighted with filled, as opposed to open diamonds.

It is important to understand how the metabolite identification information content of the metabolites varies with their structures. Fig. 4 shows the variation in the number of bits of metabolite identification information against the number of carbon atoms in the molecule. An approximately linear relationship is observed, apart from three clear outliers (filled diamonds in Fig. 4) due to the sugars xylose, fucose and glucose in the set. Removal of the three outliers improves the linear correlation to an R^2^ of 0.47, with the equation y = 1.74 x  − 0.48.

**Fig. 5:**
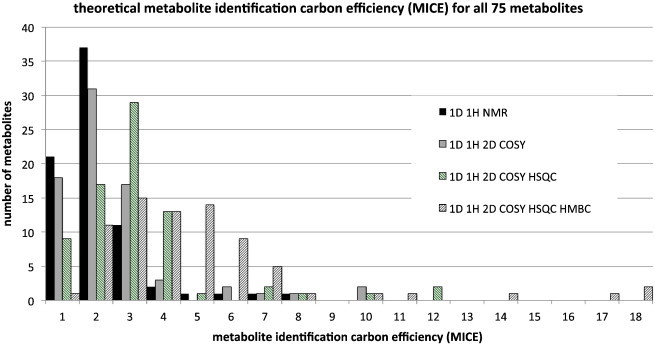
The theoretical metabolite identification carbon efficiency (MICE) for all 75 metabolites and for four separate metabolic profiling approaches: 1D ^1^H NMR alone, 1D ^1^H plus COSY, 1D ^1^H plus COSY and HSQC data and 1D ^1^H plus COSY, HSQC and HMBC data. The histogram shows the number of metabolites for each approach with MICE values in bins of 0 to 1, > 1 to 2, > 2 to 3 etc. up to > 17 to18.

The analysis was then developed using a concept from drug discovery. In 2004, Alex, Groom and Hopkins introduced the concept of ligand efficiency as a tool to assist lead and drug discovery [45,46]. The essence of this approach is to calculate the binding energy of ligands *per heavy atom in the molecule*, in order to drive drug discovery projects towards molecules that have the highest binding energy with the lowest molecular weight. A corresponding approach to metabolite identification analysis would use the concept of metabolite identification efficiency (MIE). In contrast to ligand efficiency (LE) where the total molecular weight is of importance, in MIE, the number of carbon atoms in the metabolite is also of importance, as the carbon atoms carry the vast majority of the non-exchangeable hydrogen atoms observed in ^1^H NMR experiments. We thus introduce the concept of metabolite identification efficiency in two forms:

$$\begin{array}{l} MIE=\mathrm{number}\;\mathrm{of}\;\mathrm{bits}\;\mathrm{of}\;\mathrm{metabolite}\;\mathrm{identification}\;\mathrm{information}/\mathrm{number}\;\mathrm{of}\;\mathrm{heavy}\;\mathrm{atoms}\;\mathrm{in}\;\mathrm{metabolite}\\ \mathrm{MICE}=\mathrm{number}\;\mathrm{of}\;\mathrm{bits}\;\mathrm{of}\;\mathrm{metabolite}\;\mathrm{identification}\;\mathrm{information}/\mathrm{number}\;\mathrm{of}\;\mathrm{carbons}\;\mathrm{in}\;\mathrm{metabolite} \end{array}$$

where MICE is the Metabolite Identification Carbon Efficiency. Like MIE, MICE can be calculated separately for each metabolite according to the approach taken to the analysis of the metabonomics data, be that solely based on 1D ^1^H NMR, or involving significant utilisation of 2D NMR methods (Fig. 5).

It is clear that the MICE for metabolites varies broadly and that the use of additional 2D technologies including COSY, HSQC and HMBC can significantly boost the theoretical amount of metabolite identification information per carbon atom in the metabolite. The theoretical MICE values range from an average of 1.8 ± 1.3 for 1D ^1^H NMR alone, to 2.2 ± 1.7 for approaches that include COSY data, to 2.9 ± 2.1 for approaches that also include COSY and HSQC and to 4.6 ± 3.3 bits per carbon atom for approaches that include COSY, HSQC and HMBC (standard deviations).

For comparison, the theoretical MIE values range from an average of 1.0 ± 0.7 for 1D ^1^H NMR alone, to 1.2 ± 0.9 for approaches that also include COSY data, to 1.6 ± 1.1 for approaches that also include COSY and HSQC and to 2.6 ± 1.8 bits per heavy atom (standard deviations) for approaches that also include HMBC. The MIE values are naturally lower as the number of heavy atoms is approximately double the number of carbon atoms across this set of 75 metabolites.

The amount of metabolite identification information per heavy atom or per carbon atom in the metabolites is quite high and gives a perspective on what can be achieved via modern, high field NMR spectroscopy approaches to metabonomics. This efficiency-based approach is critical in understanding how much metabolite identification information is being obtained relative to the molecular size of the metabolite.

The theoretical MIE and MICE values also varied significantly according to the type of metabolites under study. The metabolites were sorted between those containing 1 to 5 chiral centres (n = 24) and those containing no chiral centres (n = 51). Table 6 shows the differences between the chiral and non-chiral metabolites in terms of their theoretical number of bits of metabolite identification information at the level of 1D ^1^H and 2D COSY and HSQC NMR data plus the corresponding MIE and MICE values.

Table 6 clearly shows that: (i) the information content of the chiral metabolites is significantly greater than that of the non-chiral metabolites (p = 0.0007), and also that (ii) the information density per heavy atom (MIE, p = 0.0022) or per carbon atom (MICE, p = 0.0014, all from two-tailed, unpaired student t-tests) is also significantly higher for chiral metabolites. In all cases the p values from the student *t*-test are less than 0.05, the cut-off for statistical significance of the differences in the values, with 95% confidence. The reason for these significant differences is principally the raising of the chemical shift degeneracy for methylene protons in the environment of a chiral centre: this significantly increases the number of metabolite identification information bits in a metabolite.

A similar theoretical analysis using an NMR approach including 1D ^1^H NMR, COSY and HSQC data was conducted of differences between the classes of metabolites in Table 4. This demonstrated that the MIE values for the tricarboxylic acids (0.8 ± 0.5, n = 4) are significantly lower than the corresponding values for the cluster formed of the small alcohols and ketones (1.9 ± 0.5, n = 5, grouped together) with a p value of 0.012. In addition the group of sugars and sugar acids have an MIE value (3.6 ± 2.1) that is significantly greater than those of all other groups (additional values are: 1.5 ± 0.4, 1.3 ± 1.1, 1.4 ± 1.0, 1.4 ± 0.6 and 1.4 ± 0.7 for the carboxylic acids, n = 11, the hydroxycarboxylic acids, n = 4, the dicarboxylic acids, n = 8, the amines, n = 14 and the amino acids and amides, n = 21 respectively) with p values all ≤ 0.039, apart from the cluster formed of the small alcohols and ketones (p = 0.079).

The corresponding theoretical MICE analysis (at the level of 1D ^1^H NMR, COSY and HSQC data) showed that the values of the sugars and sugar acids (7.1 ± 4.1) are significantly greater than those of all other groups, with p values ranging from 0.012 to 0.031. The MICE values for the other groups are 2.3 ± 0.6, 2.5 ± 1.9, 2.7 ± 1.9, 1.7 ± 1.1, 2.8 ± 0.7, 2.5 ± 1.1 and 2.6 ± 1.2 for the carboxylic acids, the hydroxycarboxylic acids, the dicarboxylic acids, the tricarboxylic acids, the small alcohols and ketones, the amines, and the amino acids and amides, respectively. No other groups showed significantly different MICE values in pairwise comparisons. The non-significance (MIE) vs the significance (MICE) in the differences between the values for the sugars and sugar acids and the small alcohols and ketones, reflects the fact that the carbon to oxygen ratio is at least 2 to 1 for the alcohols and ketones whereas it is ca 1:1 for most of the sugars and sugar acids. This has the effect of scaling down the MIE values for the sugars and sugar acids and making the difference between their average values and those of the small alcohols and ketones non-significant.

It is worth noting that a third approach, different from either the MIE or MICE approaches is possible. This third approach involves simply counting the number of bits of metabolite identification information theoretically present in each of the metabolites, with each level of NMR approach, from 1D ^1^H NMR alone, up to the combined usage of 1D ^1^H NMR together with COSY, HSQC and HMBC data. The number of theoretical metabolite ID information bits can then be compared with the actual number of bits experimentally observed to give a metabolite identification hydrogen fraction (MIHF). This analysis is conducted in Section 3.4 below.

### 3.4 An analysis of the actual 1D and 2D ^1^H NMR spectroscopic information content of metabolites, retrieved from analysis of biofluid NMR spectra

Theoretical analyses are all well and good but a key question is how much metabolite identification information is *actually retrieved* in typical metabonomics experiments. Issues such as relatively low abundance of a particular metabolite and/or spectral crowding in some chemical shift regions will reduce the actual amount of metabolite identification information retrieved for metabolites, relative to the theoretical maximal amount. In addition, the small size and lack of hydrogen atoms in some metabolites limit the amount of information available.

Table 7 lists the information obtained using four different levels of NMR spectroscopy for the 75 metabolites studied in this work. A direct comparison with Table 5 will illustrate that there is a significant drop in the amount of information obtained from the analysis of the experimental NMR spectra, compared with that which is theoretically available. Table 8 provides another view of the data, providing the total number of bits of metabolite identification information actually obtained in four different modes of NMR-based metabonomics versus the bits of information theoretically available.

The drop off in metabolite identification information observed relative to that theoretically available is particularly steep for the HMBC data. Only 82 bits of information out of a possible total of 725 bits were obtained across all 75 metabolites from HMBC experiments. This is unsurprising given the difficulty in acquiring HMBC data on low abundance metabolites in biofluids with good sensitivity in a reasonable period of time. However, for HSQC, an encouraging 129 bits of information were obtained from a theoretical maximum of 250 across the 75 metabolites.

Supplementary Fig. 3 shows a histogram comparing the actual number of metabolite identification information bits retrieved in the experiments reported here compared with the amount theoretically available, for an approach combining information from 1D ^1^H NMR, COSY and HSQC experiments. The clustering of the actual information retrieved to lower bin sizes is clear.

**Fig. 6:**
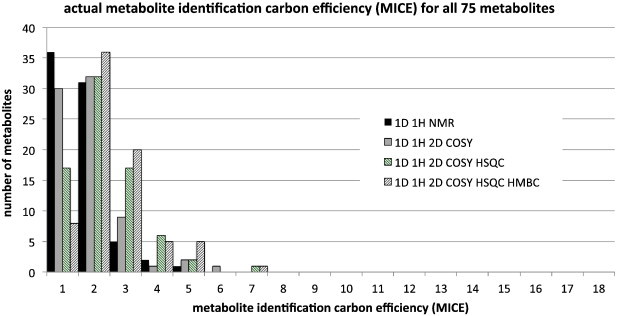
The actual experimental metabolite identification carbon efficiency (MICE) for all 75 metabolites and for four separate metabolic profiling approaches: 1D ^1^H NMR alone, 1D ^1^H plus COSY, 1D ^1^H plus COSY and HSQC data and 1D ^1^H plus COSY, HSQC and HMBC data. The histogram shows the number of metabolites for each approach with MICE values in bins of 0 to 1, > 1 to 2, > 2 to 3 etc. up to > 17 to18.

Finally, Fig. 6 shows the actual metabolite identification carbon efficiency (MICE) obtained in the experiments with four different NMR approaches.

**Fig. 7:**
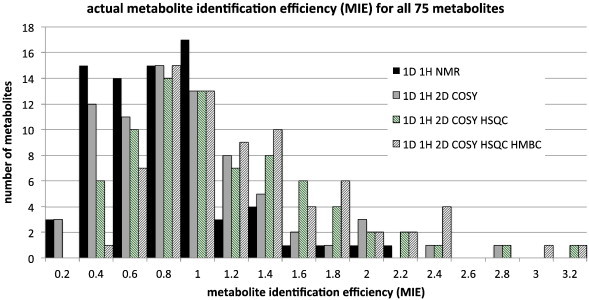
The actual experimental metabolite identification efficiency (MIE) for all 75 metabolites and for four separate metabolic profiling approaches: 1D ^1^H NMR alone, 1D ^1^H plus COSY, 1D ^1^H plus COSY and HSQC data and 1D ^1^H plus COSY, HSQC and HMBC data. The histogram shows the number of metabolites for each approach with MIE values in bins of 0 to 0.2, > 0.2 to 0.4, > 0.4 to 0.6 etc. up to > 3.0 to 3.2.

The data in Fig. 6 can be directly compared with that in Fig. 5. It is clear that the actual, experimental MICE values for metabolites vary broadly and that the use of additional 2D technologies including COSY and HSQC does boost the actual amount of metabolite identification information per carbon atom in the metabolite. However, in these experiments, the additional information from HMBC did not augment the information available to anywhere near the extent theoretically possible. The actual average MICE values over all metabolites range from 1.3 ± 0.8 for 1D ^1^H NMR alone, to 1.6 ± 1.0 for approaches that also include COSY, to 1.9 ± 1.1 for approaches that also include COSY and HSQC and to 2.2 ± 1.1 bits per carbon atom (standard deviations) for approaches that also include HMBC. The corresponding actual MIE values (Fig. 7) averaged over all 75 metabolites are: 0.7 ± 0.4 for 1D ^1^H NMR alone, 0.9 ± 0.5 for approaches that also include COSY, 1.1 ± 0.6 for approaches that also include COSY and HSQC and 1.2 ± 0.6 bits per heavy atom for approaches that also include COSY, HSQC and HMBC (standard deviations).

As mentioned in Section 3.3 above, another approach to take to the question of metabolite identification confidence would be to compare simply the number of metabolite identification information bits obtained experimentally, with the number of bits theoretically present in each metabolite to arrive at a metabolite identification hydrogen fraction (MIHF) as defined below:

$$\begin{array}{l} \mathrm{MIHF}=\mathrm{NMIIo}/\mathrm{NMIIt}\\ \mathrm{NMIIo}=\mathrm{Number}\;\mathrm{of}\;\mathrm{bits}\;\mathrm{of}\;\mathrm{Metabolite}\;\mathrm{Identification}\;\mathrm{Information}\;\mathrm{actually}\;o\mathrm{bserved}\\ \mathrm{NMIIt}=\mathrm{Number}\;\mathrm{of}\;\mathrm{bits}\;\mathrm{of}\;\mathrm{Metabolite}\;\mathrm{Identification}\;\mathrm{Information}\;t\mathrm{heoretically}\;\mathrm{present} \end{array}$$

**Fig. 8:**
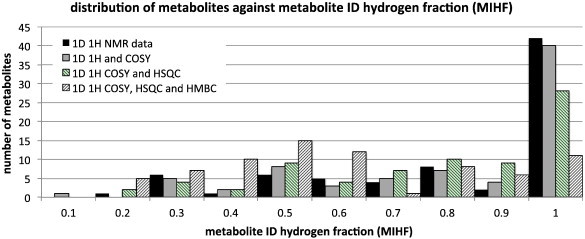
A histogram of the number of metabolites in the collection of 75 metabolites analysed here against the metabolite identification hydrogen fraction (MIHF) in buckets of 0.1 from 0 to 1. The analysis is shown for four separate NMR approaches to metabolite identification: use of 1D ^1^H NMR data alone and the additional uses of COSY, HSQC and HMBC data.

MIHF can be calculated for single metabolites, sub-groups of metabolites or an entire collection. This analysis is also illuminating (Fig. 8).

It is striking that 42 out of 75 metabolites (56%) have MIHF values of > 0.9 for a simple 1D ^1^H NMR approach to metabolite identification, indicating that the majority of metabolites studied here are displaying > 90% of the available 1D ^1^H NMR information bits. This % drops off as the NMR approach includes the use of more and more 2D NMR methods and is lowest for the approach combining 1D ^1^H NMR with 2D COSY, HSQC and HMBC approaches. This is due to the difficulty of observing all HMBC cross-peaks for metabolites present in a biofluid at relatively low concentrations.

As discussed above, the MIHF values clustered significantly at the high end of the range of possible values and provided a less good discrimination between metabolites than the corresponding MIE or MICE values. In addition, the MIHF values can seem misleadingly low for chiral metabolites, where there is typically more information than is required for confident metabolite identification, due to the raising of the degeneracy of methylene proton signals. For instance, a comparison of achiral, 2-hydroxyisobutyric acid (2-hydroxy-2-methylpropanoic acid, HMDB00729) with its chiral isomer, 3-hydroxyisobutyric acid ((2S)-3-hydroxy-2-methylpropanoic acid, HMDB00023) shows that the former has a total of just 2 bits of spectroscopic information at the level of 1D ^1^H NMR information bits, whereas the latter has 12! Finally, it is also a concern that it may be easier for a small metabolite with a low number of signals to get a very high MIHF score, compared with a more complex metabolite with more signals. Consequently, the MIE and MICE measures of confidence in metabolite identification were used in the rest of this analysis in preference to the MIHF.

### 3.5 How much NMR information is enough for confident metabolite identification?

This is the key question. The Metabolomics Standards Initiative (MSI) approach differentiates between the situation where: (i) the experimental metabonomics data is compared with an authentic reference standard (Level 1, Identified Metabolite) and (ii) where comparison is made to the literature or a public domain database such as the HMDB (Level 2, Putatively Annotated Metabolite): see Table 1. On the basis of the analysis of the NMR-derived data in this study, that differentiation is not appropriate and it is perfectly possible to confidently identify known metabolites based on reference to the literature or the public databases. The guidelines to enable this are proposed to be as follows:

1. experimental metabolite identification carbon efficiency (MICE) ideally ≥ 1 and/or metabolite identification efficiency (MIE) > 0.5, [these are guidelines, not rigid cut-offs, based on the experience with the metabolites in this study and are for MICE and MIE values with NMR approaches based on 1D ^1^H plus 2D COSY and HSQC data]
2. the fit of the experimental data to reference data should be precise, generally within ± 0.03 ppm for 1D ^1^H and ± 0.5 ppm for ^13^C NMR shifts and ± 0.2 Hz for homonuclear proton couplings: values outside these limits need explanation: in addition, the reference database entries should be double-checked for consistency with other literature values and general accuracy and self-consistency, including by downloading of actual free induction decay data e.g., from the HMDB [31]
3. the NMR spectral data should provide ‘coverage’ of all parts of the molecule: for example, for para-cresol glucuronide, a molecule with two distinct parts, it is important to have NMR data from both the cresol and glucuronide parts for good confidence
4. the signal-to-noise ratio and the resolution (actual and digital) in the spectra should be sufficient to measure the signal features with confidence, with high resolution having the added benefit of enabling the observation of long-range, homonuclear ^1^H —^1^H and two-bond ^1^H —^14^N couplings that can be diagnostic for certain metabolites
5. care needs to be applied in the assignments of signals in regions of the ^1^H NMR spectrum that are crowded with signals from other metabolites, as the possibility of miss-assignment is higher in these regions: high spectral and digital resolution is even more critical, as is the ability to correlate the correct signals together: TOCSY and J-resolved spectra can be enabling here
6. HSQC data is extremely important in resolving metabolite identification issues, as the chemical shift sensitivity of ^13^C NMR is ca 20 × that of ^1^H NMR and it provides a superb orthogonal data source, as recommended by MSI: reliance solely on 1D ^1^H NMR data will lead to confident assignments of major metabolites but will struggle with the confident identification of less prominent metabolites in crowded spectral regions
7. even though HMBC provided only a small proportion of the metabolite identification bits that were theoretically possible in these experiments, it is sometimes the only way to categorically identify metabolites. HMBC is extremely valuable for defining inter-atomic connectivities to quaternary carbon atoms, as well as through quaternary carbon atoms and heteroatoms, and should be used as much as possible.

The example of the identification of l-fucose is a good one, if on the extreme end of proving a point. A total of 15 bits of 1D ^1^H NMR information were discovered in the experimental data. This figure increased to 20, 24 and 25 bits of information if COSY, COSY plus HSQC or COSY plus HSQC and HMBC data respectively, were considered in addition to the 1D ^1^H NMR data. The experimental MICE values were 2.5, 3.3, 4.0 and 4.2 for the 1D ^1^H NMR, 1D ^1^H plus 2D COSY, 1D ^1^H plus 2D COSY and HSQC, and 1D ^1^H plus 2D COSY, HSQC and HMBC data approaches respectively. The corresponding MIE values were: 1.4, 1.8, 2.2 and 2.3 respectively. All the experimental bits of metabolite identification information were in good agreement with those reported for authentic l-fucose, HMDB00174, in the HMDB (see Section 3.1 above). These figures indicate great confidence in the metabolite identification and no need for any further direct comparisons with an actual sample of authentic l-fucose as recommended in the original MSI publication [36].

A metabolite with an MICE value just under average for approaches based on 1D ^1^H plus 2D COSY and HSQC data is ketoleucine (HMDB00695). This is a more normal example of a metabolite that was identified in the mouse urine. The methyl groups were observed as a doublet at 0.941 (d, 6.6 Hz), 24.5 with a COSY to 2.098 (triplet of septets), and the latter signal had a COSY to 2.618 (d, 7.0 Hz). The identification of three chemical shifts, three multiplicities, two coupling constants, two COSY and one HSQC cross-peaks gave a total of 11 bits of information. The corresponding data for HMDB00695 was 2.60 (d, 7.0), 50.8; 2.09, 26.7 and 0.93 (d, 6.7 Hz), 24.4 and is an excellent match to the experimental data. Ketoleucine has 6 carbon atoms, so the MICE value is 11/6 = 1.8, just under the average MICE value of 1.9 bits per carbon for all the metabolites in this study, and at this level. Ketoleucine, a metabolite with a below average MICE value is considered confidently identified.

### 3.6 How confident can we be in metabolite identification with MIE < 0.5 or MICE < 1 (using 1D ^1^H plus COSY and HSQC NMR data)?

This analysis will be exemplified for NMR approaches that use 1D ^1^H plus COSY and HSQC NMR data, as this is routine in metabonomics/metabolomics studies. Of the 75 metabolites in this study, five have a theoretical MIE of < 0.5 and four of these five have a theoretical MICE of < 1 based on combined 1D ^1^H plus COSY and HSQC NMR data (See Supplementary Data). These metabolites are: 2-hydroxyisobutyric acid, succinic acid, tartaric acid, allantoin and guanidoacetic acid. All five metabolites have just a single singlet in their 1D ^1^H NMR spectrum, severely limiting the amount of NMR information that can be obtained. In practice, the relatively distinctive chemical shifts of the first four, the availability of HSQC information for all five and HMBC information for all except 2-hydroxyisobutyric acid, means that their identification is unambiguous (see Supplementary Information). However, in these cases, where the MIE < 0.5 and/or MICE is < 1, it is critical to have orthogonal confirmation of metabolite identities via HSQC/HMBC data, as achieved in the experiments reported here, and all five metabolites are considered confidently identified. The actual experimental MICE, MIE values were: 2-hydroxyisobutyric acid (0.8, 0.4), succinic acid (0.8, 0.4), tartaric acid (0.8, 0.3), allantoin (0.8, 0.3) and guanidoacetic acid (1.0, 0.4), all being identical to the maximum theoretical values in this case.

In addition, 13 of the 75 metabolites studied have *actual* MIE scores of < 0.5 and/or actual MICE scores of < 1.0 based on the combined experimental 1D ^1^H plus COSY and HSQC NMR data (See Supplementary Data). These 13 naturally include the five metabolites analysed above. Table 9 extracts the data that was available for the MICE scores of the 8 additional metabolites from the Supplementary materials.

So, for these metabolites, how confident is their identification based on the information given in Table 9? For phenylacetic acid, three additional HMBC connectivities were observed from the acid, and ipso and ortho aromatic carbons to the methylene protons, which also had a long-range, 4-bond COSY to the ortho aromatic protons, so this identification is considered confident. For methylsuccinic acid, no additional information was available and therefore this metabolite should be described as putatively annotated, to keep consistency with the MSI nomenclature. For trans-aconitic acid, in addition to the 1D ^1^H NMR chemical shift, multiplicity and HSQC information, a long-range, 4-bond COSY was observed between the olefin and methylene protons, both of which displayed characteristic 0.8 Hz couplings on resolution enhancement of the spectra. Reprocessing the reference NMR data in HMDB (HMDB00958) with resolution enhancement reveals couplings of ca 0.7 and 0.8 Hz on the methylene and olefin signals respectively, in agreement, so this metabolite is considered confidently identified. For choline, in addition to the 1D ^1^H NMR chemical shift, multiplicity and HSQC information, cross-methyl, and N—CH_2_ carbon to methyl proton HMBC peaks were observed. Remarkably, due to the quasi-symmetrical environment of the quadrupolar nitrogen-14 atom, resolution enhancement of the methyl signal revealed a small ^2^J_NH_ of ca 0.6 Hz (triplet 1:1:1) which is diagnostic and also present on reprocessing the HMDB reference spectrum with resolution enhancement (HMDB00097). This identification is thus considered confident. For l-carnitine, in addition to the proton chemical shift and multiplicity of the methyl group, an HSQC cross-peak to the methyl carbon was observed, together with HMBC cross-peaks from the methyl carbon and the N—CH_2_ carbon to the methyl protons. This metabolite is considered putatively annotated however, as none of the metabolite identification information covers the carboxylic acid portion of the molecule (see Guideline 3 in Section 3.5 above). For dimethylglycine, in addition to the 1D ^1^H NMR chemical shift, multiplicity and HSQC information, cross-methyl and N—CH_2_ carbon to methyl HMBC peaks were observed, confirming the identification of this metabolite. For betaine, in addition to the 1D ^1^H NMR chemical shift, multiplicity and HSQC information, cross-methyl, and N—CH_2_ carbon to methyl proton HMBC peaks were observed, confirming the identification. For *N*-propionylglycine however, no further information was available and thus, this metabolite should be described as putatively annotated also.

In summary for 13 metabolites with *actual* MIE scores of < 0.5 and/or actual MICE scores of < 1.0 based on the combined experimental 1D ^1^H plus COSY and HSQC NMR data, a total of three metabolites were classed as putatively annotated: the rest were confidently identified. Thus, even with relatively low MIE or MICE scores, it is still possible to confidently identify a very large number of metabolites, *as long as additional, high quality 1D and 2D NMR data is available*.

## 4 Conclusions

This work represents a novel, more quantitative approach to the issue of confidence in metabolite identification. The spectroscopic information content of the 1D and 2D ^1^H NMR spectra of metabolites has been investigated from a metabolite identification perspective for the first time. New theoretical and experimental measures of metabolite identification efficiency have been delineated: the metabolite identification efficiency (MIE), the metabolite identification carbon efficiency (MICE) and the metabolite identification hydrogen fraction (MIHF). These are expected to be useful in helping to establish the confidence of metabolite identifications in future metabonomics/metabolomics studies.

The main recommendation emerging from this work is that the requirement for comparison with an authentic reference standard for confident metabolite identification is unnecessary for NMR-based metabolite identifications as long as the 7 recommendations for metabolite identification confidence below are acted upon (see also Section 3.5). Metabolites can be confidently identified by comparison with data in online databases such as HMDB [31]. Examples have been given of confident identifications of metabolites with high, average and relatively low MIE/MICE values, using data at the level of 1D ^1^H plus 2D COSY and HSQC data. Metabolites with low MIE/MICE values will need corroboration with other data. In the case of experiments run at the level of 1D ^1^H plus 2D COSY and HSQC data, this may be HMBC or long-range coupling data, for example.

The 7 recommendations for confident identification of known metabolites based on comparison with the NMR spectra of those metabolites in reference databases such as HMDB that emerged from this work (see Section 3.4 above for more details) are:

1. the experimental metabolite identification carbon efficiency (MICE) obtained in the experiments ideally should be ≥ 1, or the metabolite identification efficiency (MIE) > 0.5: these are guidelines, not absolute numbers, and are for approaches using 1D ^1^H plus 2D COSY and HSQC data
2. the fit of the experimental data to reference data should be precise, generally within ± 0.03 ppm for ^1^H, and ± 0.5 ppm for ^13^C NMR shifts and ± 0.2 Hz for proton couplings: the database entries should be double-checked for self-consistency, accuracy and agreement with other literature, including by downloading of actual free induction decay data (HMDB)
3. the NMR spectral data should provide ‘coverage’ of all parts of the molecule
4. the signal-to-noise ratio and the resolution (actual and digital) in the spectra should be sufficient to measure the signal features with confidence
5. care should be applied when assigning signals in crowded spectral regions
6. HSQC data is important in metabolite identification, as it provides an excellent orthogonal data source via the ^13^C NMR chemical shift
7. HMBC data should be used wherever possible to corroborate identifications.

A further recommendation from this work is that metabonomics/metabolomics researchers publish more detail on the spectroscopic data on which they are basing their metabolite identifications. This additional information could include the MIE or MICE values for each of the metabolites identified. Confidence in metabolite identification is critical for any subsequent biochemical or biological interpretation of the data.

Thus, in summary, as long as the 7 recommendations above are acted upon, confident identifications of *known metabolites* can be made by reference to on-line databases such as the Human Metabolome Database (HMDB). Out of 75 known metabolites studied in this work, it is asserted that 72 of 75 (96%) are confidently identified and only 3 metabolites (4%) fell into the putatively annotated category.

One of the reasons for being less conservative in the identification of known metabolites using NMR spectroscopic methods is that NMR technology is stable and reproducible. Having an excellent resource like the HMDB [31] available, that not only provides access to *information* on the NMR spectra of the metabolites in both 1D and 2D forms, but also enables access to the raw free induction decay data, is equivalent in many cases to having access to an authentic reference standard for direct comparisons. However, as always, the researcher needs to double-check all database entries for coherence and accuracy: errors in the databases do occur.

It is hoped that this work provides a new paradigm for NMR-based metabolite identification of *known metabolites*. It is expected that other researchers will investigate and test the methodology and no doubt develop it further. However, it is hoped and expected that the provision of a metabolite identification confidence index such as MIE or MICE will help solve the current issue of confidence in metabolite identification. Finally, it should be noted that the ideas herein are equally applicable to mass spectrometry, and to other analytical techniques, and should have broad utility.

## Figures and Tables

**Table:** 

Term	Meaning
1D	One-dimensional
2D	Two-dimensional
CAWG	Chemical Analysis Working Group
CE–MS	Capillary electrophoresis mass spectrometry
COSY	COrrelated SpectroscopY
δ_H_	Hydrogen-1 or proton NMR chemical shift
δ_C_	Carbon-13 NMR chemical shift
GC–MS	Gas chromatography mass spectrometry
HMBC	Heteronuclear multiple bond correlation spectroscopy
HMDB	Human Metabolome Database
HSQC	Heteronuclear single quantum correlation spectroscopy
ID	Identification
^3J^_H,H_	Three-bond spin–spin coupling between two hydrogens etc
JRES	J-resolved spectroscopy
LC–MS	Liquid chromatography mass spectrometry
MIE	Metabolite identification efficiency
MICE	Metabolite identification carbon efficiency
MIHF	Metabolite Identification Hydrogen Fraction
MS	Mass spectrometry
MSI	Metabolomics Standards Initiative
NOESY	Nuclear Overhauser spectroscopy
NMR	Nuclear magnetic resonance
TOCSY	TOtal Correlation SpectroscopY
TSP	Sodium 3-(trimethylsilyl) propionate-2, 2, 3, 3-d4
UPLC–MS	Ultra-performance liquid chromatography mass spectrometry

**Table 1:** The four levels of known metabolite identification from the CAWG 2007 [36].

Level 1	*Identified Compound*: A minimum of two independent and orthogonal data (such as retention time and mass spectrum) compared directly relative to an authentic reference standard
Level 2	*Putatively Annotated Compound*: Compound identified by analysis of spectral data and/or similarity to data in a public database but without direct comparison to a reference standard as for Level 1
Level 3	*Putatively Characterised Compound Class*: unidentified per se but the data available allows the metabolite to be placed in a compound class
Level 4	*Unknown Compound*: unidentified or unclassified but characterised by spectral data

**Table 2:** The 14 molecular and spectroscopic features calculated for the 75 metabolites.

1. Number of hydrogen atoms	2. Number of carbon atoms	3. Number of oxygen atoms
4. Number of nitrogen atoms	5. Number of sulphur atoms	6. Nominal mass in Da
7. Number of chiral centres	8. Number of ^1^H NMR chemical shifts	9. Number of multiplicities
10. Number of 2- or 3-bond H, H coupling constants	11. Second order flag = 0 or 1	12. Number of 2D ^1^H COSY cross-peaks
13. Number of 2D ^1^H, ^13^C HSQC cross-peaks	14. Number of 2D ^1^H, ^13^C HMBC cross-peaks	

**Table 3:** Metabolite identification parameters calculated for the 75 metabolites.

Parameter	Calculation
A. Total number of heavy atoms	Sum of features 2 to 5 in Table 2
B. Total number of spectroscopic information bits available from 1D ^1^H NMR	Sum of features 8 to 11
C. Total number of spectroscopic information bits available from 1D ^1^H and 2D ^1^H COSY NMR	Sum of features 8 to 12
D. Total number of spectroscopic information bits available from 1D ^1^H and 2D ^1^H COSY and HSQC NMR	Sum of features 8 to 13
E. Total number of spectroscopic information bits available from 1D ^1^H and 2D ^1^H COSY, HSQC and HMBC NMR	Sum of features 8 to 14
F. Theoretical metabolite identification carbon efficiency (MICE) for 1D ^1^H NMR	(Sum of features 8 to 11)/number of carbon atoms
G. Theoretical metabolite identification carbon efficiency (MICE) for 1D ^1^H and 2D ^1^H COSY NMR	(Sum of features 8 to 12)/number of carbon atoms
H. Theoretical metabolite identification carbon efficiency (MICE) for 1D ^1^H and 2D ^1^H COSY and HSQC NMR	(Sum of features 8 to 13)/number of carbon atoms
I. Theoretical metabolite identification carbon efficiency (MICE) for 1D ^1^H and 2D ^1^H COSY, HSQC and HMBC NMR	(Sum of features 8 to 14)/number of carbon atoms
J. Theoretical metabolite identification efficiency (MIE) for 1D ^1^H NMR	(Sum of features 8 to 11)/number of heavy atoms
K. Theoretical metabolite identification efficiency (MIE) for 1D ^1^H and 2D ^1^H COSY NMR	(Sum of features 8 to 12)/number of heavy atoms
L. Theoretical metabolite identification efficiency (MIE) for 1D ^1^H and 2D ^1^H COSY and HSQC NMR	(Sum of features 8 to 13)/number of heavy atoms
M. Theoretical metabolite identification efficiency (MIE) for 1D ^1^H and 2D ^1^H COSY, HSQC and HMBC NMR	(Sum of features 8 to 14)/number of heavy atoms

**Table 4:** The 75 metabolites identified by NMR spectroscopy in recent metabonomics studies on human and mouse urine.

Metabolite class	Common name	IUPAC name
Carboxylic acids	Formic acid	Formic acid
	Acetic acid	Acetic acid
	Propionic acid	Propanoic acid
	Butyric acid	Butanoic acid
	Isobutyric acid	2-Methylpropanoic acid
	Isovaleric acid	2-Methylbutanoic acid
	Ketoleucine	4-Methyl-2-oxopentanoic acid
	Benzoic acid	benzoic acid
	Phenylacetic acid	2-Phenylacetic acid
	Para-hydroxy-phenylacetic acid	2-(4-Hydroxyphenyl)acetic acid
	Hydrocinnamic acid	3-Phenylpropanoic acid
Hydroxycarboxylic acids	Glycolic acid	2-Hydroxyacetic acid
	Lactic acid	(2S)-2-hydroxypropanoic acid
	2-Hydroxyisobutyric acid	2-Hydroxy-2-methylpropanoic acid
	3-Hydroxyisobutyric acid	(2S)-3-hydroxy-2-methylpropanoic acid
Dicarboxylic acids	Succinic acid	Butanedioic acid
	l-Malic acid	(2S)-2-hydroxybutanedioic acid
	Tartaric acid	(2R,3R)-2,3-Dihydroxybutanedioic acid
	Methylsuccinic acid	2-Methylbutanedioic acid
	Glutaric acid	Pentanedioic acid
	2-Hydroxyglutaric acid	(2S)-2-hydroxypentanedioic acid
	2-Ketoglutaric acid	2-Oxopentanedioic acid
	2-Isopropylmalic acid	(2S)-2-hydroxy-2-(propan-2-yl)butanedioic acid
Tricarboxylic acid	Citric acid	2-Hydroxypropane-1,2,3-tricarboxylic acid
	Isocitric acid	1-Hydroxypropane-1,2,3-tricarboxylic acid
	cis-Aconitic acid	(1Z)-Prop-1-ene-1,2,3-tricarboxylic acid
	Trans-aconitic acid	(1E)-Prop-1-ene-1,2,3-tricarboxylic acid
Small alcohols	Ethanol	Ethanol
	Chiral 2, 3-butanediol	(2R,3R)-butane-2,3-diol or (2S,3S)-butane-2,3-diol
	Meso-2, 3-butanediol	(2R,3S)-2,3-butanediol
Ketones	Butanone	Butan-2-one
	Acetoin	3-Hydroxybutan-2-one
Sugars and sugar acids	d-Xylose	(3R,4S,5R)-oxane-2,3,4,5-tetrol
	l-Fucose	(3S,4R,5S,6S)-6-methyloxane-2,3,4,5-tetrol
	d-Glucose	(3R,4S,5S,6R)-6-(hydroxymethyl)oxane-2,3,4,5-tetrol
	Mannitol	(2R,3R,4R,5R)-hexane-1,2,3,4,5,6-hexol
	d-Glucaric acid	(2R,3S,4S,5S)-2,3,4,5-tetrahydroxyhexanedioic acid
	d-Glucuronic acid	(2S,3S,4S,5R,6S)-3,4,5,6-tetrahydroxyoxane-2-carboxylic acid
	Para-cresol glucuronide	(2S,3S,4S,5R,6S)-3,4,5-trihydroxy-6-(4-methylphenoxy)oxane-2-carboxylic acid
Amines	Methylamine	Methanamine
	Dimethylamine	Dimethylamine
	Trimethylamine	Trimethylamine
	Trimethylamine *N*-oxide	*N*,*N*-dimethylmethanamine oxide
	Ethanolamine	2-Aminoethan-1-ol
	Choline	(2-Hydroxyethyl)trimethylazanium
	3-Methylhistamine	2-(1-Methyl-1H-imidazol-5-yl)ethan-1-amine
	Hypotaurine	2-Aminoethane-1-sulfinic acid
	Taurine	2-Aminoethane-1-sulfonic acid
	3-Indoxyl sulphate	1H-indol-3-yloxidanesulfonic acid
	Putrescine	Butane-1,4-diamine
	Creatinine	2-Imino-1-methylimidazolidin-4-one
	Creatine	2-(1-Methylcarbamimidamido)acetic acid
	l-Carnitine	(3R)-3-hydroxy-4-(trimethylazaniumyl)butanoate
Amino acids and amides	Glycine	2-Aminoacetic acid
	*N*-methylglycine, sarcosine	2-(Methylamino)acetic acid
	Dimethylglycine	2-(Dimethylamino)acetic acid
	*N*,*N*,*N*-trimethylglycine, betaine	2-(Trimethylazaniumyl)acetate
	*N*-acetylglycine	2-Acetamidoacetic acid
	*N*-propionylglycine	2-Propanamidoacetic acid
	*N*-butyrylglycine	2-Butanamidoacetic acid
	*N*-isovalerylglycine	2-(3-Methylbutanamido)acetic acid
	Hippuric acid, benzoylglycine	2-(Phenylformamido)acetic acid
	Phenylacetylglycine	2-(2-Phenylacetamido)acetic acid
	Guanidoacetic acid	2-Carbamimidamidoacetic acid
	Ureidopropionic acid	3-(Carbamoylamino)propanoic acid
	l-Alanine	(2S)-2-aminopropanoic acid
	Beta-alanine	3-Aminopropanoic acid
	Pyroglutamic acid	(2S)-5-oxopyrrolidine-2-carboxylic acid
	l-Histidine	(2S)-2-amino-3-(1H-imidazol-4-yl)propanoic acid
	1-Methylhistidine	(2S)-2-amino-3-(1-methyl-1H-imidazol-4-yl)propanoic acid
	Allantoin	(2,5-Dioxoimidazolidin-4-yl)urea
	Trigonelline	1-Methylpyridin-1-ium-3-carboxylate
	1-Methylnicotinamide	3-Carbamoyl-1-methylpyridin-1-ium
	Cytosine	6-Amino-1,2-dihydropyrimidin-2-one
Other metabolites	Para-cresol sulphate	(4-Methylphenyl)oxidanesulfonic acid

**Table 5:** The number of bits of spectroscopic information per metabolite *theoretically contained* in the group of 75 metabolites, from four NMR-based metabonomics approaches: each bit corresponds to a bit of metabolite identification information.

Feature/methodology	1D ^1^H NMR	1D ^1^H NMR plus 2D COSY	1D ^1^H NMR plus 2D COSY and HSQC	1D ^1^H NMR plus 2D COSY, HSQC and HMBC
Minimum number of bits	2	2	3	3
Maximum number of bits	42	56	70	106
Average number of bits	9.2	11.3	14.7	24.3
Median number of bits	7	8	11	16
Standard deviation	7.9	10.6	13.1	21.8

**Table 6:** A theoretical analysis of the total number of metabolite identification information bits, metabolite identification efficiency (MIE) and metabolite identification carbon efficiency (MICE) for chiral (24) vs non-chiral (n = 51) metabolites in this study (all analyses at the level of data from 1D ^1^H and 2D COSY and HSQC NMR.

Feature/parameter	Average value	Standard deviation
Total number of metabolite identification information bits for chiral metabolites	24.58	17.98
Total number of metabolite identification information bits for non-chiral metabolites	9.98	6.03
Metabolite identification efficiency MIE, chiral	2.36	1.52
Metabolite identification efficiency MIE, non-chiral	1.27	0.56
Metabolite identification carbon efficiency (MICE), chiral	4.45	3.03
Metabolite identification carbon efficiency (MICE), non-chiral	2.19	0.88

**Table 7:** The bits of metabolite identification information per metabolite *actually obtained* from four NMR-based metabonomics approaches in the group of 75 metabolites.

Feature/methodology	1D ^1^H NMR	1D ^1^H NMR plus 2D COSY	1D ^1^H NMR plus 2D COSY and HSQC	1D ^1^H NMR plus 2D COSY, HSQC and HMBC
Minimum number of bits	2	2	2	2
Maximum number of bits	22	28	31	31
Average number of bits	6.2	7.5	9.2	10.3
Median number of bits	5	6	8	9
Standard deviation	4.5	5.8	6.5	6.8

**Table 8:** A comparison of the total amount of metabolite identification information actually obtained versus that theoretically available from four NMR-based metabonomics approaches across the group of 75 metabolites as a whole.

Feature/methodology	1D ^1^H NMR	1D ^1^H NMR plus 2D COSY	1D ^1^H NMR plus 2D COSY and HSQC	1D ^1^H NMR plus 2D COSY, HSQC and HMBC
Theoretical total number of metabolite identification bits available	688	849	1099	1824
Actual total number of metabolite identification bits observed	467	560	689	771

**Table 9:** the actual NMR-based metabolic identification information available from 1D ^1^H plus COSY and HSQC NMR experiments on eight metabolites with MICE scores of < 1.0.

Common name	Number of carbon atoms	Number of 1D 1H δH	Number of mult.	Number of nJHH	Actual 2nd order flag	Number of COSY cross-peaks	Number of HSQC peaks	Actual total info 1D 1H, COSY & HSQC	Actual MICE based on 1D 1H COSY HSQC
Phenylacetic acid	8	1	1	0	1	0	4	7	0.9
Methylsuccinic acid	5	1	1	1	0	1	0	4	0.8
Trans-aconitic acid	6	2	2	0	0	0	1	5	0.8
Choline	5	1	1	0	0	0	1	3	0.6
l-Carnitine	7	1	1	0	0	0	1	3	0.4
Dimethylglycine	4	1	1	0	0	0	1	3	0.8
*N*,*N*,*N*-trimethylglycine, betaine	5	1	1	0	0	0	1	3	0.6
*N*-propionylglycine	5	1	1	1	0	1	0	4	0.8

## Appendix B Supplementary Data

Supplementary Figures.

Supplementary Spreadsheet.
