# Supplementary material for: A New Paradigm for Known Metabolite Identification in Metabonomics/Metabolomics: Metabolite Identification Efficiency
Source: Comput Struct Biotechnol J. 2015 Jan 27;13:131–44. doi: 10.1016/j.csbj.2015.01.002 (PMC4348432; doi:10.1016/j.csbj.2015.01.002)
Supplement: Supplementary file 1 — Supplementary Tables. [file mmc1.pdf]

Supplementary Tables for Ms. Ref. No.: CSBJ-D-14-00051

Computational and Structural Biotechnology Journal entitled "A New Paradigm for Known Metabolite Identification in Metabonomics/ Metabolomics: Metabolite Identification Efficiency." J. R. Everett

Supplementary Table 1: Typical Data Acquisition and Processing Parameters for 2D NMR spectra.

parameter	JRES	COSY	TOCSY	HSQC	HMBC
F2 spectral width in Hz	12,019	6,009	6,128	9,615	6,203
F1 spectral width in Hz	50	6004	6,126	30,187	33,523
data points in t2	16,384	4096	2,048	1,024	2,048
spectral size in F2	32,768	8,192	4,096	4,096	4,096
increments in t1	120	256	512	400	400
spectral size in F1	1,024	4,096	4,096	2,048	2,048
number of scans	32	64	32	64	64
relaxation delay in seconds	2.00	2.00	1.50	2.00	2.00
apodisation	sine bell in t1 and t2 with first point correction in t1	sine bell squared in t2, sine square with first point correction in t1	sine bell squared in t2, sine square with first point correction in t1	sine bell squared in t2, sine square with first point correction in t1	sine bell in t1 and t2 with first point correction in t1
Bruker pulse sequence code	lcjresprqf	cosygpqfpr.ht	dipsi2esgpph	hsqcetgpprsisp2.2.be	hmbcgplpndprqf
other points	tilted and symmetrised	t1 noise reduction applied			

Supplementary Table 2: The Information Content of NMR spectra in Metabonomics/Metabolomics

#	feature	comment
1	chemical shift	• sensitive to fine details of molecular structure, including isomerism, and to the environment of the molecule: solvent, binding etc<br>• generally reproducible to ± 0.03 for ^{1}H and ± 0.5 ppm for ^{13}C NMR in biofluid spectra
2	multiplicity	• the form of the signal: singlet, doublet, doublet of doublets etc<br>• reveals the number of coupling partners for a hydrogen atom in a metabolite
3	coupling constant	• provides information on the geometric and electronic relationships between the coupling hydrogen atoms
4	1st or 2nd order	• most coupled spectra are second order to the extent that there is a slight alteration of the intensities of the lines in coupled multiplets, such that it appears that coupled signals are 'roofed' or leaning towards each other<br>• this occurs significantly when the frequency separation between the coupled signals is < 10 x $J_{H,H}$<br>• in some cases, especially where there are hydrogens that are chemically equivalent (and therefore have identical chemical shifts), but magnetically non-equivalent, the effects on signal intensities are severe and additional lines may appear in the spectra
5	signal half-bandwidth or $\nu_{1/2}$	• inversely proportional to the spin-spin relaxation time of the signal<br>• reflects the motional and exchange environment of the hydrogen atom and the molecule that it is in
6	signal integral	• directly proportional to the number of hydrogens responsible for that signal in the molecule i.e. CH vs CH_2 vs CH_3, and to the relative proportion of the molecule in the biofluid under study
7	COSY cross-peak	• automatic information on the connectivities and chemical nature (via their chemical shifts) of hydrogen atoms in the same molecule that are separated by 2 to 3 chemical bonds (generally) from a given hydrogen
8	HSQC cross-peak	• automatic information on the chemical nature (via the chemical shift) of the carbon atom in the same molecule to which a hydrogen is directly attached
9	HMBC cross-peaks	• automatic information on the connectivities and chemical nature (via their chemical shifts) of the carbon atoms in the same molecule that are separated by 2 to 3 chemical bonds (generally) from a given hydrogen
10	TOCSY cross-peaks	• automatic information on the connectivities and chemical nature (via their chemical shifts) of hydrogen atoms in the same molecule that are connected in the same spin system as a given hydrogen

11	signal stability: rate of change of integral	• rate of degradation or conversely synthesis of a molecule<br>• for example, the conversion of phosphorylcholine to choline in human seminal fluid post-ejaculation[44]
