# Supplementary material for: A New Paradigm for Known Metabolite Identification in Metabonomics/Metabolomics: Metabolite Identification Efficiency
Source: Comput Struct Biotechnol J. 2015 Jan 27;13:131–44. doi: 10.1016/j.csbj.2015.01.002 (PMC4348432; doi:10.1016/j.csbj.2015.01.002)
Supplement: Supplementary file 2 — Supplementary Figures. [file mmc2.pdf]

Supplementary Figures for Ms. Ref. No.: CSBJ-D-14-00051

Computational and Structural Biotechnology Journal entitled "A New Paradigm for Known Metabolite Identification in Metabonomics/ Metabolomics: Metabolite Identification Efficiency." J. R. Everett

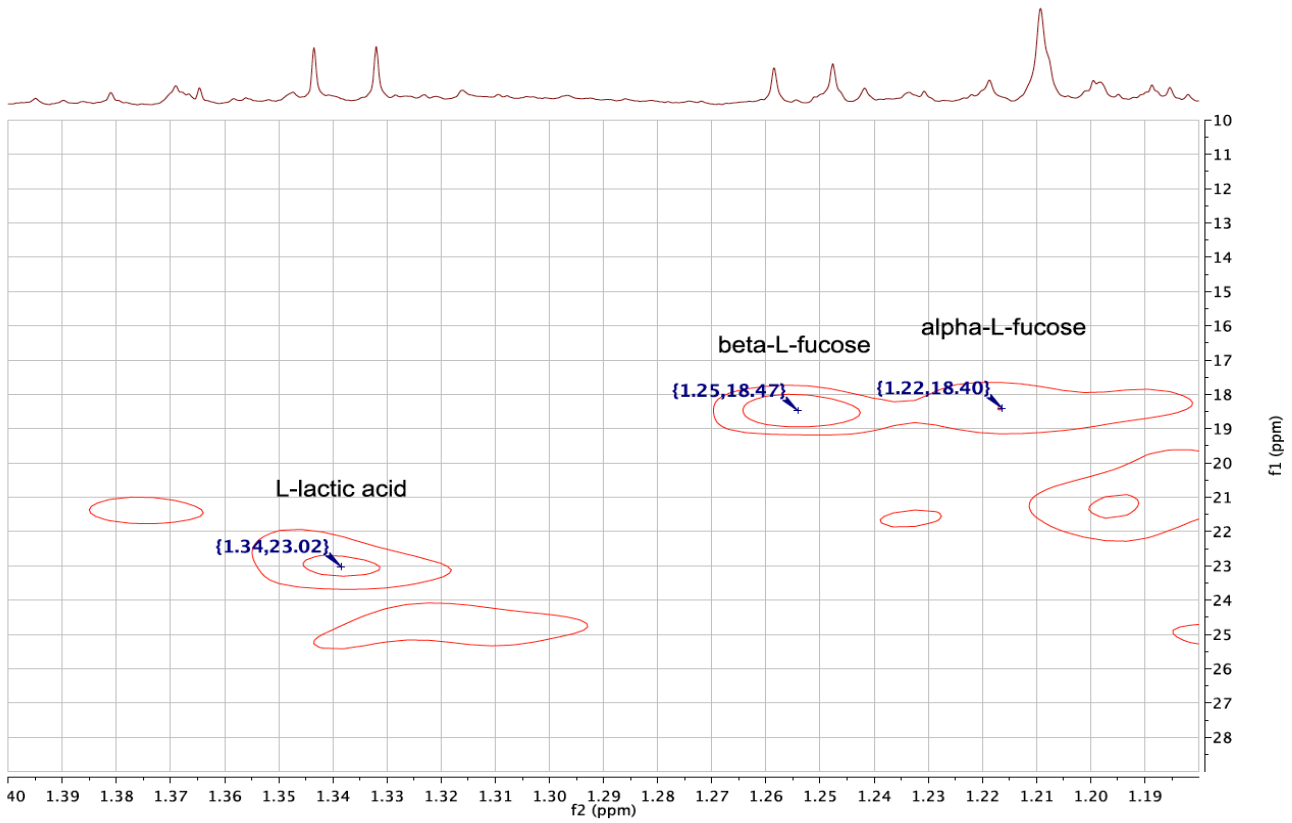

Supplementary Figure 1: an expansion of the 600 MHz 2D ^{1}H, 13C HSQC NMR spectrum of the urine from a C57BL/6 mouse in the region of the methyl signals from lactic acid and the two anomers of fucose, underneath the corresponding region of the 1D ^{1}H NMR spectrum. Proton NMR chemical shifts are along the x-axis and carbon-13 NMR chemical shifts along the y-axis. Note that the exact chemical shift for the methyl group of the alpha anomer of fucose in 1D ^{1}H NMR spectra of this urine is 1.213.

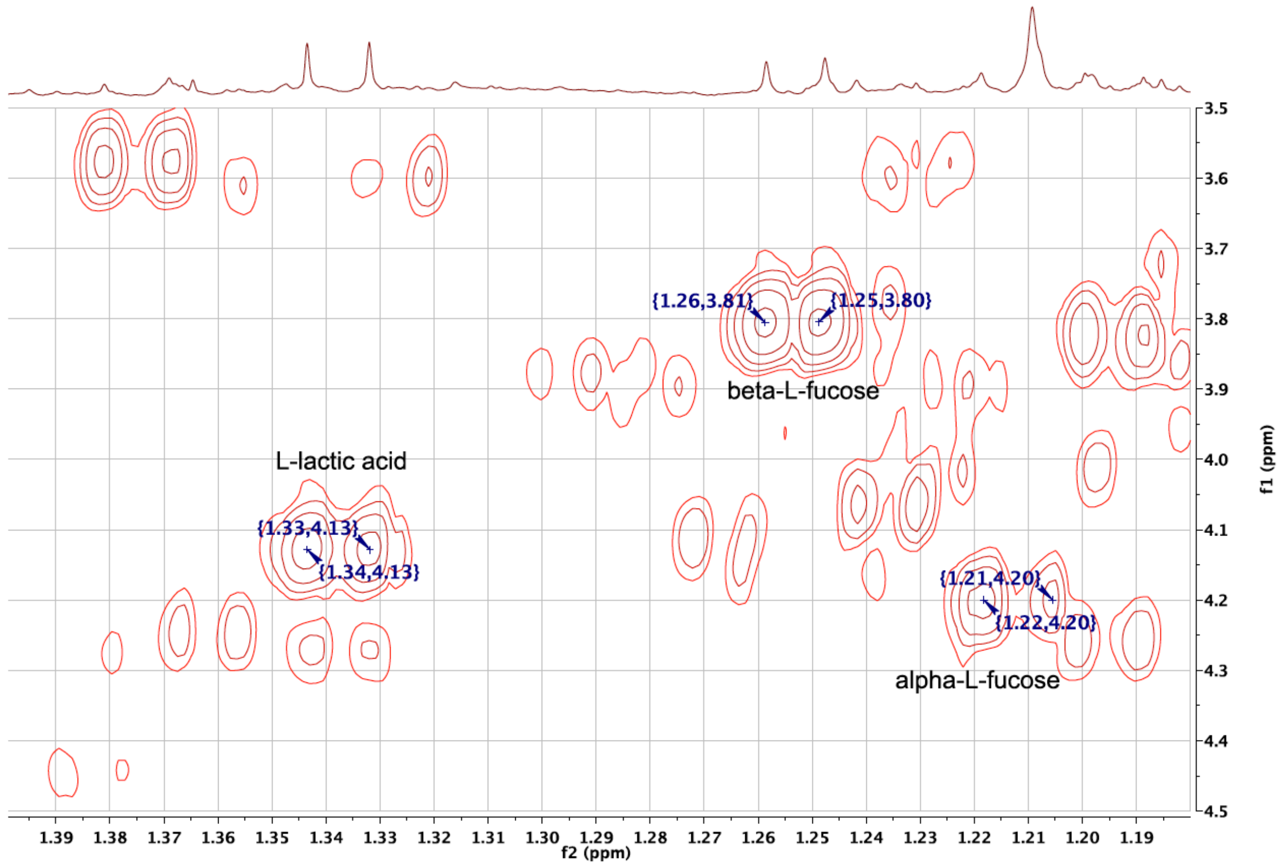

Supplementary Figure 2: an expansion of the 600 MHz 2D ^1H COSY NMR spectrum of the urine from a C57BL/6 mouse in the region of the methyl signals from lactic acid and the two anomers of fucose, underneath the corresponding region of the 1D ^1H NMR spectrum.

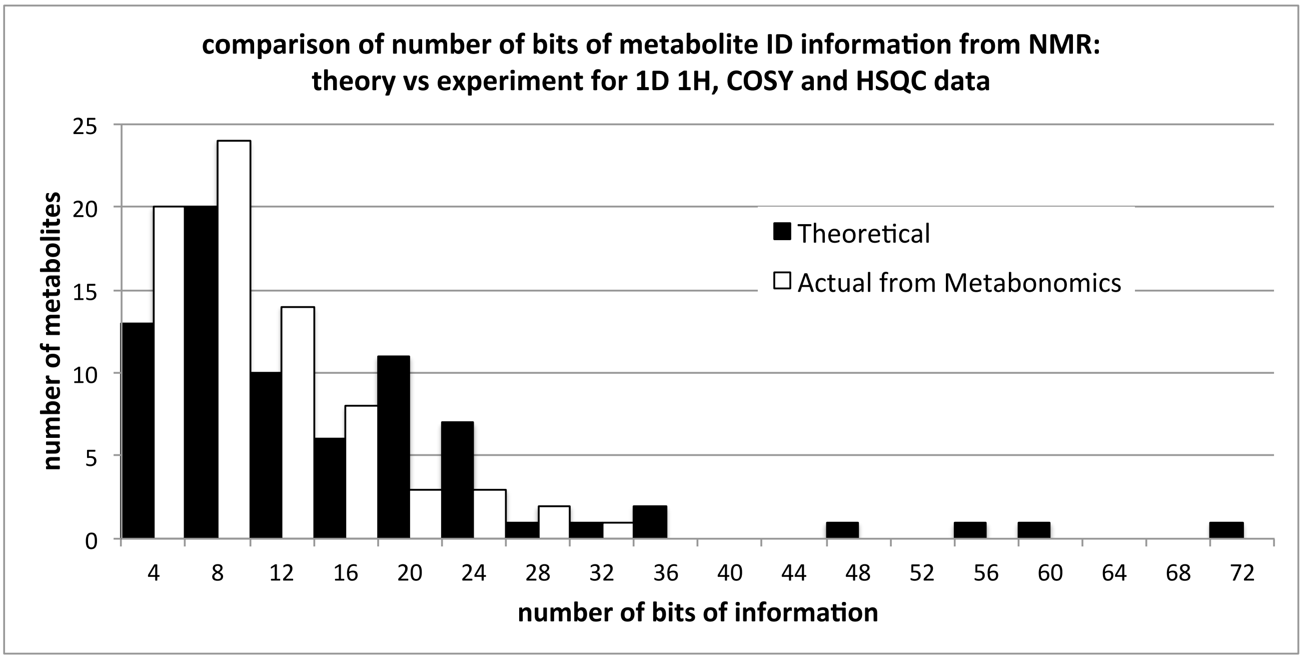

Supplementary Figure 3: a comparison of the total number of bits of metabolite identification (ID) information available from experiments combining 1D ^{1}H, COSY and HSQC NMR, compared with the number of bits of information theoretically available. The x-axis displays the number of bits available in bins from 0 to 4, 5 to 8 bits etc.
