# Supplementary material for: A New Paradigm for Known Metabolite Identification in Metabonomics/Metabolomics: Metabolite Identification Efficiency
Source: Comput Struct Biotechnol J. 2015 Jan 27;13:131–44. doi: 10.1016/j.csbj.2015.01.002 (PMC4348432; doi:10.1016/j.csbj.2015.01.002)
Supplement: Supplementary file 3 — Supplementary Spreadsheet. [file mmc3.pdf]

metabolite class	common name	IUPAC name	HMDB code	1 ♯ H	2 ♯ C	3 ♯ O	4 ♯ N	5 ♯ S	A total ♯ heavy atoms	6 nominal mass	7 ♯ chiral centres	8 ♯ 1D 1H δH	9 ♯ mult.	10 ♯ nJHH	11 2nd order flag	12 ♯ COSY	13 ♯ HSQC	14 ♯ HMBC
carboxylic acids	formic acid	formic acid	00142	2	1	2	0	0	3	46	0	1	1	0	0	0	1	0
	acetic acid	acetic acid	00042	4	2	2	0	0	4	60	0	1	1	0	0	0	1	1
	propionic acid	propionic acid	00237	6	3	2	0	0	5	74	0	2	2	1	0	1	2	4
	butyric acid	butanoic acid	00039	8	4	2	0	0	6	88	0	3	3	2	0	2	3	8
	isobutyric acid	2-methylpropanoic acid	01873	8	4	2	0	0	6	88	0	2	2	1	0	1	2	4
	isovaleric acid	2-methylbutanoic acid	00718	10	5	2	0	0	7	102	0	3	3	2	0	2	3	8
	ketoleucine	4-methyl-2-oxopentanoic acid	00695	10	6	3	0	0	9	130	0	3	3	2	0	2	3	9
	benzoic acid	benzoic acid	01870	6	7	2	0	0	9	122	0	3	3	2	1	2	3	11
	phenylacetic acid	2-phenylacetic acid	00209	8	8	2	0	0	10	136	0	4	4	2	1	2	4	14
	para-hydroxy-phenylacetic acid	2-(4-hydroxyphenyl)acetic acid	00020	8	8	3	0	0	11	152	0	3	3	1	1	1	3	12
	hydrocinnamic acid	3-phenylpropanoic acid	00764	10	9	2	0	0	11	150	0	5	5	3	1	3	5	18
hydroxycarboxylic acids	glycolic acid	2-hydroxyacetic acid	03035	4	2	3	0	0	5	76	0	1	1	0	0	0	1	1
	lactic acid	(2S)-2-hydroxypropanoic acid	00190	6	3	3	0	0	6	90	1	2	2	1	0	1	2	4
	2-hydroxyisobutyric acid	2-hydroxy-2-methylpropanoic acid	00729	8	4	3	0	0	7	104	0	1	1	0	0	0	1	1
	3-hydroxyisobutyric acid	(2S)-3-hydroxy-2-methylpropanoic acid	00023	8	4	3	0	0	7	104	1	4	4	4	0	4	4	12
dicarboxylic acids	succinic acid	butanedioic acid	00254	6	4	4	0	0	8	118	0	1	1	0	0	0	1	2
	L-malic acid	(2S)-2-hydroxybutanedioic acid	00156	6	4	5	0	0	9	134	1	3	3	3	0	3	3	9
	tartaric acid	(2R,3R)-2,3-dihydroxybutanedioic acid	00956	6	4	6	0	0	10	150	2	1	1	0	0	0	1	2
	methylsuccinic acid	2-methylbutanedioic acid	01844	8	5	4	0	0	9	132	1	4	4	4	0	4	4	15
	glutaric acid	pentanedioic acid	00661	8	5	4	0	0	9	132	0	2	2	1	0	1	2	5
	2-hydroxyglutaric acid	(2S)-2-hydroxypentanedioic acid	00694	8	5	5	0	0	10	148	1	5	5	8	0	8	5	17
	2-ketoglutaric acid	2-oxopentanedioic acid	00208	6	5	5	0	0	10	146	0	2	2	1	0	1	2	7
	2-isopropylmalic acid	(2S)-2-hydroxy-2-(propan-2-yl)butanedioic acid	00402	12	7	5	0	0	12	176	1	5	5	3	0	3	5	19
tricarboxylic acids	citric acid	2-hydroxypropane-1,2,3-tricarboxylic acid	00094	8	6	7	0	0	13	192	0	2	2	1	0	1	2	8
	isocitric acid	1-hydroxypropane-1,2,3-tricarboxylic acid	00193	8	6	7	0	0	13	192	2	4	4	4	0	4	4	17
	cis-aconitic acid	(1Z)-prop-1-ene-1,2,3-tricarboxylic acid	00072	6	6	6	0	0	12	174	0	2	2	0	0	0	2	8
	trans-aconitic acid	(1E)-prop-1-ene-1,2,3-tricarboxylic acid	00958	6	6	6	0	0	12	174	0	2	2	0	0	0	2	8
small alcohols	ethanol	ethanol	00108	6	2	1	0	0	3	46	0	2	2	1	0	1	2	2
	chiral 2, 3-butanediol	(2R,3R)-butane-2,3-diol or (2S,3S)-butane-2,3-diol	33007	10	4	2	0	0	6	90	2	2	2	1	1	1	2	3
	meso-2, 3-butanediol	(2R,3S)-2,3-Butanediol	03156	10	4	2	0	0	6	90	2	2	2	1	1	1	2	3
ketones	butanone	butan-2-one	00474	8	4	1	0	0	5	72	0	3	3	1	0	1	3	7
	acetoin	3-hydroxybutan-2-one	03243	8	4	2	0	0	6	88	1	3	3	1	0	1	3	7
sugars and sugar acids	D-xylose	(3R,4S,5R)-oxane-2,3,4,5-tetrol	00098	10	5	5	0	0	10	150	4	12	12	12	0	12	12	26
	L-fucose	(3S,4R,5S,6S)-6-methyloxane-2,3,4,5-tetrol	00174	12	6	5	0	0	11	164	5	12	12	10	0	10	12	42
	D-glucose	(3R,4S,5S,6R)-6-(hydroxymethyl)oxane-2,3,4,5-tetrol	00122	12	6	6	0	0	12	180	5	14	14	14	0	14	14	36
	mannitol	(2R,3R,4R,5R)-hexane-1,2,3,4,5,6-hexol	00765	14	6	6	0	0	12	182	4	4	4	4	0	4	4	11
	D-glucuronic acid	(2S,3S,4S,5R,6S)-3,4,5,6-tetrahydroxyoxane-2-carboxylic acid	00127	10	6	7	0	0	13	194	5	10	10	8	1	8	10	36
	D-glucaric acid	(2R,3S,4S,5S)-2,3,4,5-tetrahydroxyhexanedioic acid	00663	10	6	8	0	0	14	210	4	4	4	3	0	3	4	14
	para-cresol glucuronide	(2S,3S,4S,5R,6S)-3,4,5-trihydroxy-6-(4-methylphenoxy)oxane-2-carboxylic acid	11686	16	13	7	0	0	20	284	5	8	8	5	1	5	8	37
amines	methylamine	methanamine	00164	5	1	0	1	0	2	31	0	1	1	0	0	0	1	0
	dimethylamine	dimethylamine	00087	7	2	0	1	0	3	45	0	1	1	0	0	0	1	1
	trimethylamine	trimethylamine	00906	9	3	0	1	0	4	59	0	1	1	0	0	0	1	1
	trimethylamine *N*-oxide	*N*,*N*-dimethylmethanamine oxide	00925	9	3	1	1	0	5	75	0	1	1	0	0	0	1	1
	ethanolamine	2-aminoethan-1-ol	00149	7	2	1	1	0	4	61	0	2	2	1	0	1	2	2
	choline	(2-hydroxyethyl)trimethylazanium	00097	14	5	1	1	0	7	104	0	3	3	1	0	1	3	5
	3-methylhistamine	2-(1-methyl-1H-imidazol-5-yl)ethan-1-amine	01861	11	6	0	3	0	9	125	0	5	5	1	0	1	5	13
	hypotaurine	2-aminoethane-1-sulfinic acid	00965	7	2	2	1	1	6	109	0	2	2	1	0	1	2	2
	taurine	2-aminoethane-1-sulfonic acid	00251	7	2	3	1	1	7	125	0	2	2	1	0	1	2	2
	3-indoxyl sulphate	1H-indol-3-yloxidanesulfonic acid	00682	7	8	4	1	1	14	213	0	5	5	3	0	3	5	20
	putrescine	butane-1,4-diamine	01414	12	4	0	2	0	6	88	0	2	2	2	0	2	2	5
	creatinine	2-imino-1-methylimidazolidin-4-one	00562	7	4	1	3	0	8	113	0	2	2	0	0	0	2	5
	creatine	2-(1-methylcarbamimidamido)acetic acid	00064	9	4	2	3	0	9	131	0	2	2	0	0	0	2	5
	L-carnitine	(3R)-3-hydroxy-4-(trimethylazaniumyl)butanoate	00062	15	7	3	1	0	11	161	1	6	6	5	0	5	6	17
amino acids and amides	glycine	2-aminoacetic acid	00123	5	2	2	1	0	5	75	0	1	1	0	0	0	1	1
	N-methylglycine, sarcosi	2-(methylamino)acetic acid	00271	7	3	2	1	0	6	89	0	2	2	0	0	0	2	3
	dimethylglycine	2-(dimethylamino)acetic acid	00092	9	4	2	1	0	7	103	0	2	2	0	0	0	2	4
	N,*N*,*N*-trimethylglycine,	2-(trimethylazaniumyl)acetate	00043	11	5	2	1	0	8	117	0	2	2	0	0	0	2	4
	N-acetylglycine	2-acetamidoacetic acid	00532	7	4	3	1	0	8	117	0	3	3	1	0	1	2	3
	N-propionylglycine	2-propanamidoacetic acid	00783	9	5	3	1	0	9	131	0	4	4	2	0	2	3	6
	N-butyrylglycine	2-butanamidoacetic acid	00808	11	6	3	1	0	10	145	0	5	5	3	0	3	4	10
	N-isovalerylglycine	2-(3-methylbutanamido)acetic acid	00678	13	7	3	1	0	11	159	0	5	5	3	0	3	4	10
	hippuric acid, benzoylglycine	2-(phenylformamido)acetic acid	00714	9	9	3	1	0	13	179	0	5	5	3	1	3	4	13
	phenylacetylglycine	2-(2-phenylacetamido)acetic acid	00821	11	10	3	1	0	14	193	0	6	6	3	1	3	5	18
	guanidoacetic acid	2-carbamimidamidoacetic acid	00128	7	3	2	3	0	8	117	0	1	1	0	0	0	1	2
	ureidopropionic acid	3-(carbamoylamino)propanoic acid	00026	8	4	3	2	0	9	132	0	3	3	2	0	2	2	5
	L-alanine	(2S)-2-aminopropanoic acid	00161	7	3	2	1	0	6	89	1	2	2	1	0	1	2	4
	beta-alanine	3-aminopropanoic acid	00056	7	3	2	1	0	6	89	0	2	2	1	0	1	2	4
	pyroglutamic acid	(2S)-5-oxopyrrolidine-2-carboxylic acid	00267	7	5	3	1	0	9	129	1	6	6	8	0	8	5	18
	L-histidine	(2S)-2-amino-3-(1H-imidazol-4-yl)propanoic acid	03412	9	6	2	3	0	11	155	1	5	5	3	0	3	5	16
	1-methylhistidine	(2S)-2-amino-3-(1-methyl-1H-imidazol-4-yl)propanoic acid	00001	11	7	2	3	0	12	169	1	6	6	3	0	3	6	20
	allantoin	(2,5-dioxoimidazolidin-4-yl)urea	00462	6	4	3	4	0	11	158	1	1	1	0	0	0	1	3
	trigonelline	1-methylpyridin-1-ium-3-carboxylate	00875	7	7	2	1	0	10	137	0	5	5	2	0	2	5	19
	1-methylnicotinamide	3-carbamoyl-1-methylpyridin-1-ium	00699	9	7	1	2	0	10	137	0	5	5	2	0	2	5	19
	cytosine	6-amino-1,2-dihydropyrimidin-2-one	00630	5	4	1	3	0	8	111	0	2	2	1	0	1	2	5
other metabolites	para-cresol sulphate	(4-methylphenyl)oxidanesulfonic acid	11635	8	7	4	0	1	12	188	0	3	3	1	1	1	3	11

common name	B total info 1D 1H	C total info 1D 1H & COSY	D total info 1D 1H COSY & HSQC	E total info 1D 1H COSY HSQC HMBC	F theor. MICE 1D 1H	G theor. MICE 1D 1H COSY	H theor. MICE 1D 1H COSY HSQC	I theor. MICE 1D 1H COSY HSQC HMBC	J theor. MIE 1D 1H	K theor. MIE 1D 1H & COSY	L theor. MIE 1D 1H COSY HSQC	M theor. MIE 1D 1H COSY HSQC HMBC	8' actual ♯ 1D 1H δH	9' actual ♯ mult.	10' actual ♯ nJHH	11' actual 2nd order flag	12' actual ♯ COSY	13' actual ♯ HSQC	14' actual ♯ HMBC	actual ♯ other ID info
formic acid	2	2	3	3	2.0	2.0	3.0	3.0	0.7	0.7	1.0	1.0	1	1	0	0	0	0	0	0
acetic acid	2	2	3	4	1.0	1.0	1.5	2.0	0.5	0.5	0.8	1.0	1	1	0	0	0	1	1	0
propionic acid	5	6	8	12	1.7	2.0	2.7	4.0	1.0	1.2	1.6	2.4	2	2	1	0	1	0	0	0
butyric acid	8	10	13	21	2.0	2.5	3.3	5.3	1.3	1.7	2.2	3.5	3	3	2	0	2	0	0	0
isobutyric acid	5	6	8	12	1.3	1.5	2.0	3.0	0.8	1.0	1.3	2.0	1	1	1	0	1	1	0	0
isovaleric acid	8	10	13	21	1.6	2.0	2.6	4.2	1.1	1.4	1.9	3.0	2	2	2	0	2	1	3	1
ketoleucine	8	10	13	22	1.3	1.7	2.2	3.7	0.9	1.1	1.4	2.4	3	3	2	0	2	1	0	1
benzoic acid	9	11	14	25	1.3	1.6	2.0	3.6	1.0	1.2	1.6	2.8	3	3	2	1	2	3	6	0
phenylacetic acid	11	13	17	31	1.4	1.6	2.1	3.9	1.1	1.3	1.7	3.1	1	1	0	1	0	4	3	1
para-hydroxy-phenylacetic acid	8	9	12	24	1.0	1.1	1.5	3.0	0.7	0.8	1.1	2.2	2	2	1	1	1	3	0	2
hydrocinnamic acid	14	17	22	40	1.6	1.9	2.4	4.4	1.3	1.5	2.0	3.6	2	2	1	1	1	2	2	1
glycolic acid	2	2	3	4	1.0	1.0	1.5	2.0	0.4	0.4	0.6	0.8	1	1	0	0	0	1	0	0
lactic acid	5	6	8	12	1.7	2.0	2.7	4.0	0.8	1.0	1.3	2.0	2	2	1	0	1	1	2	0
2-hydroxyisobutyric acid	2	2	3	4	0.5	0.5	0.8	1.0	0.3	0.3	0.4	0.6	1	1	0	0	0	1	0	0
3-hydroxyisobutyric acid	12	16	20	32	3.0	4.0	5.0	8.0	1.7	2.3	2.9	4.6	1	1	1	0	1	1	0	0
succinic acid	2	2	3	5	0.5	0.5	0.8	1.3	0.3	0.3	0.4	0.6	1	1	0	0	0	1	2	0
L-malic acid	9	12	15	24	2.3	3.0	3.8	6.0	1.0	1.3	1.7	2.7	2	2	3	0	2	1	0	0
tartaric acid	2	2	3	5	0.5	0.5	0.8	1.3	0.2	0.2	0.3	0.5	1	1	0	0	0	1	1	0
methylsuccinic acid	12	16	20	35	2.4	3.2	4.0	7.0	1.3	1.8	2.2	3.9	1	1	1	0	1	0	0	0
glutaric acid	5	6	8	13	1.0	1.2	1.6	2.6	0.6	0.7	0.9	1.4	2	1	1	0	1	2	0	0
2-hydroxyglutaric acid	18	26	31	48	3.6	5.2	6.2	9.6	1.8	2.6	3.1	4.8	3	3	2	0	4	1	0	1
2-ketoglutaric acid	5	6	8	15	1.0	1.2	1.6	3.0	0.5	0.6	0.8	1.5	2	2	1	0	1	2	0	0
2-isopropylmalic acid	13	16	21	40	1.9	2.3	3.0	5.7	1.1	1.3	1.8	3.3	3	3	2	0	2	5	0	0
citric acid	5	6	8	16	0.8	1.0	1.3	2.7	0.4	0.5	0.6	1.2	2	2	1	0	1	2	6	0
isocitric acid	12	16	20	37	2.0	2.7	3.3	6.2	0.9	1.2	1.5	2.8	4	4	4	0	4	4	0	0
cis-aconitic acid	4	4	6	14	0.7	0.7	1.0	2.3	0.3	0.3	0.5	1.2	2	2	0	0	0	2	4	1
trans-aconitic acid	4	4	6	14	0.7	0.7	1.0	2.3	0.3	0.3	0.5	1.2	2	2	0	0	0	1	0	1
ethanol	5	6	8	10	2.5	3.0	4.0	5.0	1.7	2.0	2.7	3.3	2	2	1	0	1	2	1	0
chiral 2, 3-butanediol	6	7	9	12	1.5	1.8	2.3	3.0	1.0	1.2	1.5	2.0	2	2	1	1	1	2	1	0
meso-2, 3-butanediol	6	7	9	12	1.5	1.8	2.3	3.0	1.0	1.2	1.5	2.0	2	2	1	1	1	2	1	0
butanone	7	8	11	18	1.8	2.0	2.8	4.5	1.4	1.6	2.2	3.6	3	3	1	0	1	1	0	0
acetoin	7	8	11	18	1.8	2.0	2.8	4.5	1.2	1.3	1.8	3.0	2	2	1	0	1	2	0	0
D-xylose	36	48	60	86	7.2	9.6	12.0	17.2	3.6	4.8	6.0	8.6	7	7	8	0	6	3	0	0
L-fucose	34	44	56	98	5.7	7.3	9.3	16.3	3.1	4.0	5.1	8.9	5	5	5	0	5	4	1	0
D-glucose	42	56	70	106	7.0	9.3	11.7	17.7	3.5	4.7	5.8	8.8	7	7	8	0	5	1	0	0
mannitol	12	16	20	31	2.0	2.7	3.3	5.2	1.0	1.3	1.7	2.6	4	3	4	0	1	4	0	0
D-glucuronic acid	29	37	47	83	4.8	6.2	7.8	13.8	2.2	2.8	3.6	6.4	7	6	6	1	5	2	0	2
D-glucaric acid	11	14	18	32	1.8	2.3	3.0	5.3	0.8	1.0	1.3	2.3	4	4	2	0	2	4	0	0
para-cresol glucuronide	22	27	35	72	1.7	2.1	2.7	5.5	1.1	1.4	1.8	3.6	5	5	3	0	2	0	0	1
methylamine	2	2	3	3	2.0	2.0	3.0	3.0	1.0	1.0	1.5	1.5	1	1	0	0	0	1	0	0
dimethylamine	2	2	3	4	1.0	1.0	1.5	2.0	0.7	0.7	1.0	1.3	1	1	0	0	0	1	1	0
trimethylamine	2	2	3	4	0.7	0.7	1.0	1.3	0.5	0.5	0.8	1.0	1	1	0	0	0	1	1	0
trimethylamine *N*-oxide	2	2	3	4	0.7	0.7	1.0	1.3	0.4	0.4	0.6	0.8	1	1	0	0	0	1	1	0
ethanolamine	5	6	8	10	2.5	3.0	4.0	5.0	1.3	1.5	2.0	2.5	2	2	1	0	1	2	0	0
choline	7	8	11	16	1.4	1.6	2.2	3.2	1.0	1.1	1.6	2.3	1	1	0	0	0	1	2	1
3-methylhistamine	11	12	17	30	1.8	2.0	2.8	5.0	1.2	1.3	1.9	3.3	3	3	0	0	0	2	0	1
hypotaurine	5	6	8	10	2.5	3.0	4.0	5.0	0.8	1.0	1.3	1.7	2	2	1	0	1	0	0	0
taurine	5	6	8	10	2.5	3.0	4.0	5.0	0.7	0.9	1.1	1.4	2	2	1	0	1	2	2	0
3-indoxyl sulphate	13	16	21	41	1.6	2.0	2.6	5.1	0.9	1.1	1.5	2.9	5	5	3	0	3	5	8	3
putrescine	6	8	10	15	1.5	2.0	2.5	3.8	1.0	1.3	1.7	2.5	2	2	1	0	1	2	0	0
creatinine	4	4	6	11	1.0	1.0	1.5	2.8	0.5	0.5	0.8	1.4	2	2	0	0	0	2	4	1
creatine	4	4	6	11	1.0	1.0	1.5	2.8	0.4	0.4	0.7	1.2	2	2	0	0	0	2	5	0
L-carnitine	17	22	28	45	2.4	3.1	4.0	6.4	1.5	2.0	2.5	4.1	1	1	0	0	0	1	2	0
glycine	2	2	3	4	1.0	1.0	1.5	2.0	0.4	0.4	0.6	0.8	1	1	0	0	0	1	1	0
N-methylglycine, sarcosi	4	4	6	9	1.3	1.3	2.0	3.0	0.7	0.7	1.0	1.5	2	2	0	0	0	0	0	0
dimethylglycine	4	4	6	10	1.0	1.0	1.5	2.5	0.6	0.6	0.9	1.4	1	1	0	0	0	1	2	0
N,*N*,*N*-trimethylglycine,	4	4	6	10	0.8	0.8	1.2	2.0	0.5	0.5	0.8	1.3	1	1	0	0	0	1	2	0
N-acetylglycine	7	8	10	13	1.8	2.0	2.5	3.3	0.9	1.0	1.3	1.6	2	2	1	0	1	1	0	0
N-propionylglycine	10	12	15	21	2.0	2.4	3.0	4.2	1.1	1.3	1.7	2.3	1	1	1	0	1	0	0	0
N-butyrylglycine	13	16	20	30	2.2	2.7	3.3	5.0	1.3	1.6	2.0	3.0	3	3	2	0	2	2	0	0
N-isovalerylglycine	13	16	20	30	1.9	2.3	2.9	4.3	1.2	1.5	1.8	2.7	2	2	2	0	2	2	3	0
hippuric acid, benzoylglycine	14	17	21	34	1.6	1.9	2.3	3.8	1.1	1.3	1.6	2.6	5	5	3	1	3	4	8	1
phenylacetylglycine	16	19	24	42	1.6	1.9	2.4	4.2	1.1	1.4	1.7	3.0	3	3	2	1	2	3	1	0
guanidoacetic acid	2	2	3	5	0.7	0.7	1.0	1.7	0.3	0.3	0.4	0.6	1	1	0	0	0	1	2	0
ureidopropionic acid	8	10	12	17	2.0	2.5	3.0	4.3	0.9	1.1	1.3	1.9	2	2	2	0	1	2	1	0
L-alanine	5	6	8	12	1.7	2.0	2.7	4.0	0.8	1.0	1.3	2.0	2	2	1	0	1	1	0	0
beta-alanine	5	6	8	12	1.7	2.0	2.7	4.0	0.8	1.0	1.3	2.0	2	2	1	0	1	0	0	0
pyroglutamic acid	20	28	33	51	4.0	5.6	6.6	10.2	2.2	3.1	3.7	5.7	2	2	1	0	2	1	0	1
L-histidine	13	16	21	37	2.2	2.7	3.5	6.2	1.2	1.5	1.9	3.4	5	5	2	0	3	5	0	5
1-methylhistidine	15	18	24	44	2.1	2.6	3.4	6.3	1.3	1.5	2.0	3.7	3	3	0	0	0	3	0	5
allantoin	2	2	3	6	0.5	0.5	0.8	1.5	0.2	0.2	0.3	0.5	1	1	0	0	0	1	2	0
trigonelline	12	14	19	38	1.7	2.0	2.7	5.4	1.2	1.4	1.9	3.8	5	3	2	0	2	5	0	3
1-methylnicotinamide	12	14	19	38	1.7	2.0	2.7	5.4	1.2	1.4	1.9	3.8	5	5	2	0	2	0	0	4
cytosine	5	6	8	13	1.3	1.5	2.0	3.3	0.6	0.8	1.0	1.6	2	1	1	0	1	0	0	0
para-cresol sulphate	8	9	12	23	1.1	1.3	1.7	3.3	0.7	0.8	1.0	1.9	3	3	1	1	1	3	0	1

common name	B' actual total info 1D 1H	C' actual total info 1D 1H & COSY	D' actual total info 1D 1H, COSY & HSQC	E' actual total info 1D 1H COSY HSQC HMBC	F' actual MICE 1D 1H	G' actual MICE 1D 1H COSY	H' actual MICE 1D 1H COSY HSQC	I' actual MICE 1D 1H COSY HSQC HMBC	J' actual MIE 1D 1H	K' actual MIE 1D 1H COSY	L' actual MIE 1D 1H COSY HSQC	M' actual MIE 1D 1H COSY HSQC HMBC	N' actual MIHF 1D 1H	O' actual MIHF 1D 1H COSY	P' actual MIHF 1D 1H COSY HSQC	R' actual MIHF 1D 1H COSY HSQC HMBC	actual total info All
formic acid	2	2	2	2	2.0	2.0	2.0	2.0	0.7	0.7	0.7	0.7	1.0	1.0	0.7	0.7	2
acetic acid	2	2	3	4	1.0	1.0	1.5	2.0	0.5	0.5	0.8	1.0	1.0	1.0	1.0	1.0	4
propionic acid	5	6	6	6	1.7	2.0	2.0	2.0	1.0	1.2	1.2	1.2	1.0	1.0	0.8	0.5	6
butyric acid	8	10	10	10	2.0	2.5	2.5	2.5	1.3	1.7	1.7	1.7	1.0	1.0	0.8	0.5	10
isobutyric acid	3	4	5	5	0.8	1.0	1.3	1.3	0.5	0.7	0.8	0.8	0.6	0.7	0.6	0.4	5
isovaleric acid	6	8	9	12	1.2	1.6	1.8	2.4	0.9	1.1	1.3	1.7	0.8	0.8	0.7	0.6	13
ketoleucine	8	10	11	11	1.3	1.7	1.8	1.8	0.9	1.1	1.2	1.2	1.0	1.0	0.8	0.5	12
benzoic acid	9	11	14	20	1.3	1.6	2.0	2.9	1.0	1.2	1.6	2.2	1.0	1.0	1.0	0.8	20
phenylacetic acid	3	3	7	10	0.4	0.4	0.9	1.3	0.3	0.3	0.7	1.0	0.3	0.2	0.4	0.3	11
para-hydroxy-phenylacetic acid	6	7	10	10	0.8	0.9	1.3	1.3	0.5	0.6	0.9	0.9	0.8	0.8	0.8	0.4	12
hydrocinnamic acid	6	7	9	11	0.7	0.8	1.0	1.2	0.5	0.6	0.8	1.0	0.4	0.4	0.4	0.3	12
glycolic acid	2	2	3	3	1.0	1.0	1.5	1.5	0.4	0.4	0.6	0.6	1.0	1.0	1.0	0.8	3
lactic acid	5	6	7	9	1.7	2.0	2.3	3.0	0.8	1.0	1.2	1.5	1.0	1.0	0.9	0.8	9
2-hydroxyisobutyric acid	2	2	3	3	0.5	0.5	0.8	0.8	0.3	0.3	0.4	0.4	1.0	1.0	1.0	0.8	3
3-hydroxyisobutyric acid	3	4	5	5	0.8	1.0	1.3	1.3	0.4	0.6	0.7	0.7	0.3	0.3	0.3	0.2	5
succinic acid	2	2	3	5	0.5	0.5	0.8	1.3	0.3	0.3	0.4	0.6	1.0	1.0	1.0	1.0	5
L-malic acid	7	9	10	10	1.8	2.3	2.5	2.5	0.8	1.0	1.1	1.1	0.8	0.8	0.7	0.4	10
tartaric acid	2	2	3	4	0.5	0.5	0.8	1.0	0.2	0.2	0.3	0.4	1.0	1.0	1.0	0.8	4
methylsuccinic acid	3	4	4	4	0.6	0.8	0.8	0.8	0.3	0.4	0.4	0.4	0.3	0.3	0.2	0.1	4
glutaric acid	4	5	7	7	0.8	1.0	1.4	1.4	0.4	0.6	0.8	0.8	0.8	0.8	0.9	0.5	7
2-hydroxyglutaric acid	8	12	13	13	1.6	2.4	2.6	2.6	0.8	1.2	1.3	1.3	0.4	0.5	0.4	0.3	14
2-ketoglutaric acid	5	6	8	8	1.0	1.2	1.6	1.6	0.5	0.6	0.8	0.8	1.0	1.0	1.0	0.5	8
2-isopropylmalic acid	8	10	15	15	1.1	1.4	2.1	2.1	0.7	0.8	1.3	1.3	0.6	0.6	0.7	0.4	15
citric acid	5	6	8	14	0.8	1.0	1.3	2.3	0.4	0.5	0.6	1.1	1.0	1.0	1.0	0.9	14
isocitric acid	12	16	20	20	2.0	2.7	3.3	3.3	0.9	1.2	1.5	1.5	1.0	1.0	1.0	0.5	20
cis-aconitic acid	4	4	6	10	0.7	0.7	1.0	1.7	0.3	0.3	0.5	0.8	1.0	1.0	1.0	0.7	11
trans-aconitic acid	4	4	5	5	0.7	0.7	0.8	0.8	0.3	0.3	0.4	0.4	1.0	1.0	0.8	0.4	6
ethanol	5	6	8	9	2.5	3.0	4.0	4.5	1.7	2.0	2.7	3.0	1.0	1.0	1.0	0.9	9
chiral 2, 3-butanediol	6	7	9	10	1.5	1.8	2.3	2.5	1.0	1.2	1.5	1.7	1.0	1.0	1.0	0.8	10
meso-2, 3-butanediol	6	7	9	10	1.5	1.8	2.3	2.5	1.0	1.2	1.5	1.7	1.0	1.0	1.0	0.8	10
butanone	7	8	9	9	1.8	2.0	2.3	2.3	1.4	1.6	1.8	1.8	1.0	1.0	0.8	0.5	9
acetoin	5	6	8	8	1.3	1.5	2.0	2.0	0.8	1.0	1.3	1.3	0.7	0.8	0.7	0.4	8
D-xylose	22	28	31	31	4.4	5.6	6.2	6.2	2.2	2.8	3.1	3.1	0.6	0.6	0.5	0.4	31
L-fucose	15	20	24	25	2.5	3.3	4.0	4.2	1.4	1.8	2.2	2.3	0.4	0.5	0.4	0.3	25
D-glucose	22	27	28	28	3.7	4.5	4.7	4.7	1.8	2.3	2.3	2.3	0.5	0.5	0.4	0.3	28
mannitol	11	12	16	16	1.8	2.0	2.7	2.7	0.9	1.0	1.3	1.3	0.9	0.8	0.8	0.5	16
D-glucuronic acid	20	25	27	27	3.3	4.2	4.5	4.5	1.5	1.9	2.1	2.1	0.7	0.7	0.6	0.3	29
D-glucaric acid	10	12	16	16	1.7	2.0	2.7	2.7	0.7	0.9	1.1	1.1	0.9	0.9	0.9	0.5	16
para-cresol glucuronide	13	15	15	15	1.0	1.2	1.2	1.2	0.7	0.8	0.8	0.8	0.6	0.6	0.4	0.2	16
methylamine	2	2	3	3	2.0	2.0	3.0	3.0	1.0	1.0	1.5	1.5	1.0	1.0	1.0	1.0	3
dimethylamine	2	2	3	4	1.0	1.0	1.5	2.0	0.7	0.7	1.0	1.3	1.0	1.0	1.0	1.0	4
trimethylamine	2	2	3	4	0.7	0.7	1.0	1.3	0.5	0.5	0.8	1.0	1.0	1.0	1.0	1.0	4
trimethylamine *N*-oxide	2	2	3	4	0.7	0.7	1.0	1.3	0.4	0.4	0.6	0.8	1.0	1.0	1.0	1.0	4
ethanolamine	5	6	8	8	2.5	3.0	4.0	4.0	1.3	1.5	2.0	2.0	1.0	1.0	1.0	0.8	8
choline	2	2	3	5	0.4	0.4	0.6	1.0	0.3	0.3	0.4	0.7	0.3	0.3	0.3	0.3	6
3-methylhistamine	6	6	8	8	1.0	1.0	1.3	1.3	0.7	0.7	0.9	0.9	0.5	0.5	0.5	0.3	9
hypotaurine	5	6	6	6	2.5	3.0	3.0	3.0	0.8	1.0	1.0	1.0	1.0	1.0	0.8	0.6	6
taurine	5	6	8	10	2.5	3.0	4.0	5.0	0.7	0.9	1.1	1.4	1.0	1.0	1.0	1.0	10
3-indoxyl sulphate	13	16	21	29	1.6	2.0	2.6	3.6	0.9	1.1	1.5	2.1	1.0	1.0	1.0	0.7	32
putrescine	5	6	8	8	1.3	1.5	2.0	2.0	0.8	1.0	1.3	1.3	0.8	0.8	0.8	0.5	8
creatinine	4	4	6	10	1.0	1.0	1.5	2.5	0.5	0.5	0.8	1.3	1.0	1.0	1.0	0.9	11
creatine	4	4	6	11	1.0	1.0	1.5	2.8	0.4	0.4	0.7	1.2	1.0	1.0	1.0	1.0	11
L-carnitine	2	2	3	5	0.3	0.3	0.4	0.7	0.2	0.2	0.3	0.5	0.1	0.1	0.1	0.1	5
glycine	2	2	3	4	1.0	1.0	1.5	2.0	0.4	0.4	0.6	0.8	1.0	1.0	1.0	1.0	4
N-methylglycine, sarcosi	4	4	4	4	1.3	1.3	1.3	1.3	0.7	0.7	0.7	0.7	1.0	1.0	0.7	0.4	4
dimethylglycine	2	2	3	5	0.5	0.5	0.8	1.3	0.3	0.3	0.4	0.7	0.5	0.5	0.5	0.5	5
N,*N*,*N*-trimethylglycine,	2	2	3	5	0.4	0.4	0.6	1.0	0.3	0.3	0.4	0.6	0.5	0.5	0.5	0.5	5
N-acetylglycine	5	6	7	7	1.3	1.5	1.8	1.8	0.6	0.8	0.9	0.9	0.7	0.8	0.7	0.5	7
N-propionylglycine	3	4	4	4	0.6	0.8	0.8	0.8	0.3	0.4	0.4	0.4	0.3	0.3	0.3	0.2	4
N-butyrylglycine	8	10	12	12	1.3	1.7	2.0	2.0	0.8	1.0	1.2	1.2	0.6	0.6	0.6	0.4	12
N-isovalerylglycine	6	8	10	13	0.9	1.1	1.4	1.9	0.5	0.7	0.9	1.2	0.5	0.5	0.5	0.4	13
hippuric acid, benzoylglycine	14	17	21	29	1.6	1.9	2.3	3.2	1.1	1.3	1.6	2.2	1.0	1.0	1.0	0.9	30
phenylacetylglycine	9	11	14	15	0.9	1.1	1.4	1.5	0.6	0.8	1.0	1.1	0.6	0.6	0.6	0.4	15
guanidoacetic acid	2	2	3	5	0.7	0.7	1.0	1.7	0.3	0.3	0.4	0.6	1.0	1.0	1.0	1.0	5
ureidopropionic acid	6	7	9	10	1.5	1.8	2.3	2.5	0.7	0.8	1.0	1.1	0.8	0.7	0.8	0.6	10
L-alanine	5	6	7	7	1.7	2.0	2.3	2.3	0.8	1.0	1.2	1.2	1.0	1.0	0.9	0.6	7
beta-alanine	5	6	6	6	1.7	2.0	2.0	2.0	0.8	1.0	1.0	1.0	1.0	1.0	0.8	0.5	6
pyroglutamic acid	5	7	8	8	1.0	1.4	1.6	1.6	0.6	0.8	0.9	0.9	0.3	0.3	0.2	0.2	9
L-histidine	12	15	20	20	2.0	2.5	3.3	3.3	1.1	1.4	1.8	1.8	0.9	0.9	1.0	0.5	25
1-methylhistidine	6	6	9	9	0.9	0.9	1.3	1.3	0.5	0.5	0.8	0.8	0.4	0.3	0.4	0.2	14
allantoin	2	2	3	5	0.5	0.5	0.8	1.3	0.2	0.2	0.3	0.5	1.0	1.0	1.0	0.8	5
trigonelline	10	12	17	17	1.4	1.7	2.4	2.4	1.0	1.2	1.7	1.7	0.8	0.9	0.9	0.4	20
1-methylnicotinamide	12	14	14	14	1.7	2.0	2.0	2.0	1.2	1.4	1.4	1.4	1.0	1.0	0.7	0.4	18
cytosine	4	5	5	5	1.0	1.3	1.3	1.3	0.5	0.6	0.6	0.6	0.8	0.8	0.6	0.4	5
para-cresol sulphate	8	9	12	12	1.1	1.3	1.7	1.7	0.7	0.8	1.0	1.0	1.0	1.0	1.0	0.5	13

common name	SMILES	InChI Code	InChI Key
formic acid	OC=O	InChI=1S/CH2O2/c2-1-3/h1H,(H,2,3)	BDAGIHXWWSANSR-UHFFFAOYSA-N
acetic acid	CC(O)=O	InChI=1S/C2H4O2/c1-2(3)4/h1H3,(H,3,4)	QTBSBXVTEAMEQO-UHFFFAOYSA-N
propionic acid	CCC(O)=O	InChI=1S/C3H6O2/c1-2-3(4)5/h2H2,1H3,(H,4,5)	XBDQKXXYIPTUBI-UHFFFAOYSA-N
butyric acid	CCCC(O)=O	InChI=1S/C4H8O2/c1-2-3-4(5)6/h2-3H2,1H3,(H,5,6)	FERIUCNNQQJTOY-UHFFFAOYSA-N
isobutyric acid	CC(C)C(O)=O	InChI=1S/C4H8O2/c1-3(2)4(5)6/h3H,1-2H3,(H,5,6)	KQNPFQTWMSNSAP-UHFFFAOYSA-N
isovaleric acid	CC(C)CC(O)=O	InChI=1S/C5H10O2/c1-4(2)3-5(6)7/h4H,3H2,1-2H3,(H,6,7)	GWYFCOCPABKNJV-UHFFFAOYSA-N
ketoleucine	CC(C)CC(=O)C(O)=O	InChI=1S/C6H10O3/c1-4(2)3-5(7)6(8)9/h4H,3H2,1-2H3,(H,8,9)	BKAJNAXTPSGJCU-UHFFFAOYSA-N
benzoic acid	c1ccc(cc1)C(=O)O	InChI=1S/C7H6O2/c8-7(9)6-4-2-1-3-5-6/h1-5H,(H,8,9)	WPYMKLBDIGXBTP-UHFFFAOYSA-N
phenylacetic acid	c1ccc(cc1)CC(=O)O	InChI=1S/C8H8O2/c9-8(10)6-7-4-2-1-3-5-7/h1-5H,6H2,(H,9,10)	WLJVXDMOQOGPHL-UHFFFAOYSA-N
para-hydroxy-phenylacetic acid	c1cc(ccc1CC(=O)O)O	InChI=1S/C8H8O3/c9-7-3-1-6(2-4-7)5-8(10)11/h1-4,9H,5H2,(H,10,11)	XQXPVVBIMDBYFF-UHFFFAOYSA-N
hydrocinnamic acid	c1ccc(cc1)CCC(=O)O	InChI=1S/C9H10O2/c10-9(11)7-6-8-4-2-1-3-5-8/h1-5H,6-7H2,(H,10,11)	XMIIGOLPHOKFCH-UHFFFAOYSA-N
glycolic acid	OCC(O)=O	InChI=1S/C2H4O3/c3-1-2(4)5/h3H,1H2,(H,4,5)	AEMRFAOFKBGASW-UHFFFAOYSA-N
lactic acid	C[C@@H](C(=O)O)O	InChI=1S/C3H6O3/c1-2(4)3(5)6/h2,4H,1H3,(H,5,6)/t2-/m0/s1	JVTAAEKCZFNVCJ-REOHCLBHSA-N
2-hydroxyisobutyric acid	CC(C)(O)C(O)=O	InChI=1S/C4H8O3/c1-4(2,7)3(5)6/h7H,1-2H3,(H,5,6)	BWLBGMIXKSTLSX-UHFFFAOYSA-N
3-hydroxyisobutyric acid	C[C@@H](CO)C(O)=O	InChI=1S/C4H8O3/c1-3(2-5)4(6)7/h3,5H,2H2,1H3,(H,6,7)/t3-/m0/s1	DBXBTMSZEOQQDU-VKHMYHEASA-N
succinic acid	OC(=O)CCC(O)=O	InChI=1S/C4H6O4/c5-3(6)1-2-4(7)8/h1-2H2,(H,5,6)(H,7,8)	KDYFGRWQOYBRFD-UHFFFAOYSA-N
L-malic acid	O[C@@H](CC(O)=O)C(O)=O	InChI=1S/C4H6O5/c5-2(4(8)9)1-3(6)7/h2,5H,1H2,(H,6,7)(H,8,9)/t2-/m0/s1	BJEPYKJPYRNKOW-REOHCLBHSA-N
tartaric acid	O[C@H]([C@@H](O)C(O)=O)C(O)=O	InChI=1S/C4H6O6/c5-1(3(7)8)2(6)4(9)10/h1-2,5-6H,(H,7,8)(H,9,10)/t1-,2-/m1/s1	FEWJPZIEWOKRBE-JCYAYHJZSA-N
methylsuccinic acid	CC(CC(O)=O)C(O)=O	InChI=1S/C5H8O4/c1-3(5(8)9)2-4(6)7/h3H,2H2,1H3,(H,6,7)(H,8,9)	WXUAQHNMJWJLTG-UHFFFAOYSA-N
glutaric acid	OC(=O)CCCC(O)=O	InChI=1S/C5H8O4/c6-4(7)2-1-3-5(8)9/h1-3H2,(H,6,7)(H,8,9)	JFCQEDHGNNZCLN-UHFFFAOYSA-N
2-hydroxyglutaric acid	O[C@@H](CCC(O)=O)C(O)=O	InChI=1S/C5H8O5/c6-3(5(9)10)1-2-4(7)8/h3,6H,1-2H2,(H,7,8)(H,9,10)/t3-/m0/s1	HWXBTNAVRSUOJR-VKHMYHEASA-N
2-ketoglutaric acid	OC(=O)CCC(=O)C(O)=O	InChI=1S/C5H6O5/c6-3(5(9)10)1-2-4(7)8/h1-2H2,(H,7,8)(H,9,10)	KPGXRSRHYNQIFN-UHFFFAOYSA-N
2-isopropylmalic acid	CC(C)[C@@](O)(CC(O)=O)C(O)=O	InChI=1S/C7H12O5/c1-4(2)7(12,6(10)11)3-5(8)9/h4,12H,3H2,1-2H3,(H,8,9)(H,10,11)/t7-/m0/s1	BITYXLXUCSKTJS-ZETCQYMHSA-N
citric acid	OC(=O)CC(O)(CC(O)=O)C(O)=O	InChI=1S/C6H8O7/c7-3(8)1-6(13,5(11)12)2-4(9)10/h13H,1-2H2,(H,7,8)(H,9,10)(H,11,12)	KRKNYBCHXYNGOX-UHFFFAOYSA-N
isocitric acid	OC(C(CC(O)=O)C(O)=O)C(O)=O	InChI=1S/C6H8O7/c7-3(8)1-2(5(10)11)4(9)6(12)13/h2,4,9H,1H2,(H,7,8)(H,10,11)(H,12,13)	ODBLHEXUDAPZAU-UHFFFAOYSA-N
cis-aconitic acid	OC(=O)C\C(=C\C(O)=O)C(O)=O	InChI=1S/C6H6O6/c7-4(8)1-3(6(11)12)2-5(9)10/h1H,2H2,(H,7,8)(H,9,10)(H,11,12)/b3-1-	GTZCVFVGUGFEME-IWQZZHSRSA-N
trans-aconitic acid	OC(=O)C\C(=C/C(O)=O)C(O)=O	InChI=1S/C6H6O6/c7-4(8)1-3(6(11)12)2-5(9)10/h1H,2H2,(H,7,8)(H,9,10)(H,11,12)/b3-1+	GTZCVFVGUGFEME-HNQUOIGGSA-N
ethanol	CCO	InChI=1S/C2H6O/c1-2-3/h3H,2H2,1H3	LFQSCWFLJHTTHZ-UHFFFAOYSA-N
chiral 2, 3-butanediol	C[C@H]([C@@H](C)O)O	InChI=1S/C4H10O2/c1-3(5)4(2)6/h3-6H,1-2H3/t3-,4-/m1/s1	OWBTYPJTUOEWEK-QWWZWVQMSA-N
meso-2, 3-butanediol	C[C@H]([C@H](C)O)O	InChI=1S/C4H10O2/c1-3(5)4(2)6/h3-6H,1-2H3/t3-,4+	OWBTYPJTUOEWEK-ZXZARUISSA-N
butanone	CCC(C)=O	InChI=1S/C4H8O/c1-3-4(2)5/h3H2,1-2H3	ZWEHNKRNPOVVGH-UHFFFAOYSA-N
acetoin	CC(O)C(C)=O	InChI=1S/C4H8O2/c1-3(5)4(2)6/h3,5H,1-2H3	ROWKJAVDOGWPAT-UHFFFAOYSA-N
D-xylose	O[C@@H]1COC(O)[C@H](O)[C@H]1O	InChI=1S/C5H10O5/c6-2-1-10-5(9)4(8)3(2)7/h2-9H,1H2/t2-,3+,4-,5?/m1/s1	SRBFZHDQGSBBOR-IOVATXLUSA-N
L-fucose	C[C@@H]1OC(O)[C@@H](O)[C@H](O)[C@@H]1O	InChI=1S/C6H12O5/c1-2-3(7)4(8)5(9)6(10)11-2/h2-10H,1H3/t2-,3+,4+,5-,6?/m0/s1	SHZGCJCMOBCMKK-DHVFOXMCSA-N
D-glucose	OC[C@H]1OC(O)[C@H](O)[C@@H](O)[C@@H]1O	InChI=1S/C6H12O6/c7-1-2-3(8)4(9)5(10)6(11)12-2/h2-11H,1H2/t2-,3-,4+,5-,6?/m1/s1	WQZGKKKJIJFFOK-GASJEMHNSA-N
mannitol	OC[C@@H](O)[C@@H](O)[C@H](O)[C@H](O)CO	InChI=1S/C6H14O6/c7-1-3(9)5(11)6(12)4(10)2-8/h3-12H,1-2H2/t3-,4-,5-,6-/m1/s1	FBPFZTCFMRRESA-KVTDHHQDSA-N
D-glucuronic acid	[C@@H]1([C@@H]([C@H]([C@@H]O[C@H]([C@@H]1O)O)C(=O)O)O)O)O	InChI=1S/C6H10O7/c7-1-2(8)4(5(10)11)13-6(12)3(1)9/h1-4,6-9,12H,(H,10,11)/t1-,2-,3+,4-,6+/m0/s1	AEMOLEFTQBMNLQ-QIUUJYRFSA-N
D-glucaric acid	O[C@@H]([C@H](O)[C@@H](O)C(O)=O)[C@H](O)C(O)=O	InChI=1S/C6H10O8/c7-1(3(9)5(11)12)2(8)4(10)6(13)14/h1-4,7-10H,(H,11,12)(H,13,14)/t1-,2-,3-,4+/m0/s1	DSLZVSRJTYRBFB-LLEIAEIESA-N
para-cresol glucuronide	Cc1ccc(cc1)O[C@H]2[C@@H]([C@H]([C@@H]([C@H](O2)C(=O)O)O)O)O	InChI=1S/C13H16O7/c1-6-2-4-7(5-3-6)19-13-10(16)8(14)9(15)11(20-13)12(17)18/h2-5,8-11,13-16H,1H3,(H,17,18)/t8-,9-,10+,11-,13+/m0/s1	JPAUCQAJHLSMQW-XPORZQOISA-N
methylamine	CN	InChI=1S/CH5N/c1-2/h2H2,1H3	BAVYZALUXZFZLV-UHFFFAOYSA-N
dimethylamine	CNC	InChI=1S/C2H7N/c1-3-2/h3H,1-2H3	ROSDSFDQCJNGOL-UHFFFAOYSA-N
trimethylamine	CN(C)C	InChI=1S/C3H9N/c1-4(2)3/h1-3H3	GETQZCLCWQTVFV-UHFFFAOYSA-N
trimethylamine *N*-oxide	C[N+](C)(C)[O-]	InChI=1S/C3H9NO/c1-4(2,3)5/h1-3H3	UYPYRKYUKCHHIB-UHFFFAOYSA-N
ethanolamine	NCCO	InChI=1S/C2H7NO/c3-1-2-4/h4H,1-3H2	HZAXFHJVJLSVMW-UHFFFAOYSA-N
choline	C[N+](C)(C)CCO	InChI=1S/C5H14NO/c1-6(2,3)4-5-7/h7H,4-5H2,1-3H3/q+1	OEYIOHPDSNJKLS-UHFFFAOYSA-N
3-methylhistamine	Cn1cncc1CCN	InChI=1S/C6H11N3/c1-9-5-8-4-6(9)2-3-7/h4-5H,2-3,7H2,1H3	CPAGZVLINCPJEH-UHFFFAOYSA-N
hypotaurine	NCCS(O)=O	InChI=1S/C2H7NO2S/c3-1-2-6(4)5/h1-3H2,(H,4,5)	VVIUBCNYACGLLV-UHFFFAOYSA-N
taurine	NCCS(O)(=O)=O	InChI=1S/C2H7NO3S/c3-1-2-7(4,5)6/h1-3H2,(H,4,5,6)	XOAAWQZATWQOTB-UHFFFAOYSA-N
3-indoxyl sulphate	c1ccc2c(c1)c(c[nH]2)OS(=O)(=O)O	InChI=1S/C8H7NO4S/c10-14(11,12)13-8-5-9-7-4-2-1-3-6(7)8/h1-5,9H,(H,10,11,12)	BXFFHSIDQOFMLE-UHFFFAOYSA-N
putrescine	NCCCCN	InChI=1S/C4H12N2/c5-3-1-2-4-6/h1-6H2	KIDHWZJUCRJVML-UHFFFAOYSA-N
creatinine	CN1CC(=O)NC1=N	InChI=1S/C4H7N3O/c1-7-2-3(8)6-4(7)5/h2H2,1H3,(H2,5,6,8)	DDRJAANPRJIHGJ-UHFFFAOYSA-N
creatine	CN(CC(O)=O)C(N)=N	InChI=1S/C4H9N3O2/c1-7(4(5)6)2-3(8)9/h2H2,1H3,(H3,5,6)(H,8,9)	CVSVTCORWBXHQV-UHFFFAOYSA-N
L-carnitine	C[N+](C)(C)C[C@H](O)CC([O-])=O	InChI=1S/C7H15NO3/c1-8(2,3)5-6(9)4-7(10)11/h6,9H,4-5H2,1-3H3/t6-/m1/s1	PHIQHXFUZVPYII-ZCFIWIBFSA-N
glycine	NCC(O)=O	InChI=1S/C2H5NO2/c3-1-2(4)5/h1,3H2,(H,4,5)	DHMQDGOQFOQNFH-UHFFFAOYSA-N
N-methylglycine, sarcosi	CNCC(O)=O	InChI=1S/C3H7NO2/c1-4-2-3(5)6/h4H,2H2,1H3,(H,5,6)	FSYKKLYZXJSNPZ-UHFFFAOYSA-N
dimethylglycine	CN(C)CC(O)=O	InChI=1S/C4H9NO2/c1-5(2)3-4(6)7/h3H2,1-2H3,(H,6,7)	FFDGPVCHZBVARC-UHFFFAOYSA-N
N,*N*,*N*-trimethylglycine,	C[N+](C)(C)CC([O-])=O	nChI=1S/C5H11NO2/c1-6(2,3)4-5(7)8/h4H2,1-3H3	KWIUHFFTVRNATP-UHFFFAOYSA-N
N-acetylglycine	CC(=O)NCC(O)=O	InChI=1S/C4H7NO3/c1-3(6)5-2-4(7)8/h2H2,1H3,(H,5,6)(H,7,8)	OKJIRPAQVSHGFK-UHFFFAOYSA-N
N-propionylglycine	CCC(=O)NCC(O)=O	InChI=1S/C5H9NO3/c1-2-4(7)6-3-5(8)9/h2-3H2,1H3,(H,6,7)(H,8,9)	WOMAZEJKVZLLFE-UHFFFAOYSA-N
N-butyrylglycine	CCCC(=O)NCC(O)=O	InChI=1S/C6H11NO3/c1-2-3-5(8)7-4-6(9)10/h2-4H2,1H3,(H,7,8)(H,9,10)	WPSSBBPLVMTKRN-UHFFFAOYSA-N
N-isovalerylglycine	CC(C)CC(=O)NCC(O)=O	InChI=1S/C7H13NO3/c1-5(2)3-6(9)8-4-7(10)11/h5H,3-4H2,1-2H3,(H,8,9)(H,10,11)	ZRQXMKMBBMNNQC-UHFFFAOYSA-N
hippuric acid, benzoylglycine	c1ccc(cc1)C(=O)NCC(=O)O	InChI=1S/C9H9NO3/c11-8(12)6-10-9(13)7-4-2-1-3-5-7/h1-5H,6H2,(H,10,13)(H,11,12)	QIAFMBKCNZACKA-UHFFFAOYSA-N
phenylacetylglycine	c1ccc(cc1)CC(=O)NCC(=O)O	InChI=1S/C10H11NO3/c12-9(11-7-10(13)14)6-8-4-2-1-3-5-8/h1-5H,6-7H2,(H,11,12)(H,13,14)	UTYVDVLMYQPLQB-UHFFFAOYSA-N
guanidoacetic acid	NC(=N)NCC(O)=O	InChI=1S/C3H7N3O2/c4-3(5)6-1-2(7)8/h1H2,(H,7,8)(H4,4,5,6)	BPMFZUMJYQTVII-UHFFFAOYSA-N
ureidopropionic acid	NC(=O)NCCC(O)=O	InChI=1S/C4H8N2O3/c5-4(9)6-2-1-3(7)8/h1-2H2,(H,7,8)(H3,5,6,9)	JSJWCHRYRHKBBW-UHFFFAOYSA-N
L-alanine	C[C@H](N)C(O)=O	InChI=1S/C3H7NO2/c1-2(4)3(5)6/h2H,4H2,1H3,(H,5,6)/t2-/m0/s1	QNAYBMKLOCPYGJ-REOHCLBHSA-N
beta-alanine	NCCC(O)=O	InChI=1S/C3H7NO2/c4-2-1-3(5)6/h1-2,4H2,(H,5,6)	UCMIRNVEIXFBKS-UHFFFAOYSA-N
pyroglutamic acid	OC(=O)[C@@H]1CCC(=O)N1	InChI=1S/C5H7NO3/c7-4-2-1-3(6-4)5(8)9/h3H,1-2H2,(H,6,7)(H,8,9)/t3-/m0/s1	ODHCTXKNWHHXJC-VKHMYHEASA-N
L-histidine	c1c(nc[nH]1)C[C@@H](C(=O)O)N	InChI=1S/C6H9N3O2/c7-5(6(10)11)1-4-2-8-3-9-4/h2-3,5H,1,7H2,(H,8,9)(H,10,11)/t5-/m0/s1	HNDVDQJCIGZPNO-YFKPBYRVSA-N
1-methylhistidine	Cn1cc(nc1)CC(C(=O)O)N	InChI=1S/C7H11N3O2/c1-10-3-5(9-4-10)2-6(8)7(11)12/h3-4,6H,2,8H2,1H3,(H,11,12)/t6-/m0/s1	BRMWTNUJHUMWMS-LURJTMIESA-N
allantoin	NC(=O)NC1NC(=O)NC1=O	InChI=1S/C4H6N4O3/c5-3(10)6-1-2(9)8-4(11)7-1/h1H,(H3,5,6,10)(H2,7,8,9,11)	POJWUDADGALRAB-UHFFFAOYSA-N
trigonelline	C[n+]1cccc(c1)C(=O)[O-]	InChI=1S/C7H7NO2/c1-8-4-2-3-6(5-8)7(9)10/h2-5H,1H3	WWNNZCOKKKDOPX-UHFFFAOYSA-N
1-methylnicotinamide	C[n+]1cccc(c1)C(=O)N	InChI=1S/C7H8N2O/c1-9-4-2-3-6(5-9)7(8)10/h2-5H,1H3,(H-,8,10)/p+1	LDHMAVIPBRSVRG-UHFFFAOYSA-O
cytosine	c1cnc(=O)[nH]c1N	InChI=1S/C4H5N3O/c5-3-1-2-6-4(8)7-3/h1-2H,(H3,5,6,7,8)	OPTASPLRGRRNAP-UHFFFAOYSA-N
para-cresol sulphate	Cc1ccc(cc1)OS(=O)(=O)O	InChI=1S/C7H8O4S/c1-6-2-4-7(5-3-6)11-12(8,9)10/h2-5H,1H3,(H,8,9,10)	WGNAKZGUSRVWRH-UHFFFAOYSA-N

Notes

1 only non-exchangeable signals are included in the 1D 1H NMR signal count: non-exchanging amides **are counted** in the 1D 1H NMR signal count

2 the number of coupling constants nJHH is only for n = 2 or 3 and includes NH - CH coupling as well as CH - CH

3 HMBC peak count is for all possible 2JCH and 3JCH options including between pseudo-equivalent methyl groups as in TMAO and is counted for every non-equivalent hydrogen signal

4 symmetrically equivalent HSQC or HMBC cross-peaks are only counted once e.g. in succinic acid i.e. we are counting the no of peaks you would see

5 the 2nd Order Flag is set to 0 if no significant second order effects and to 1 if 2nd order effects produce additional transitions (rooofing does not count as significant)

6 for sugar metabolites the analysis counts both the signals of the alpha and beta anomers in one entry

7 all IUPAC Names, SMILES strings, InChI Codes and Keys from HMDB on 21 and 22 Nov 2014 unless otherwise noted

8 for metabolite ID efficiency, the number of carbon atoms is more important that the total number of heavy atoms, as it is the carbons that carry the non-exchangeable protons (with exception of amides etc) and therefore the 1D 1H and the HSQC information

9 for COSY analysis only count each cross-peak once: therefore the number of COSY bits of information = the number of nJHH values

10 the actual additional info column includes: long-range COSY over 4 or 5 bonds, heteronuclear coupling (NH), TOCSY info where providing additional data

11 the info from 1D 1H NMR was assisted by 2D JRES NMR: the latter adds no new information (except about coupling connectivities for 2nd order systems)
